# UV/Chlorine Treatment of Sulfamethoxazole: Removal, Transformation Products, Ecotoxicity, and Environmental Fate

**DOI:** 10.3390/molecules31183220

**Published:** 2026-09-12

**Authors:** Waldemar Studziński, Alicja Gackowska, Edyta Kudlek, Maciej Przybyłek

**Affiliations:** 1 Department of Food Analysis and Environmental Protection, Faculty of Chemical Technology and Engineering, Bydgoszcz University of Science and Technology, Seminaryjna 3, 85-326 Bydgoszcz, Poland; alicja.gackowska@pbs.edu.pl; 2 Department of Water and Wastewater Engineering, Faculty of Energy and Environmental Engineering, Silesian University of Technology, Konarskiego 18, 44-100 Gliwice, Poland; edyta.kudlek@polsl.pl; 3 Department of Physical Chemistry, Faculty of Pharmacy, Nicolaus Copernicus University in Toruń, Ludwik Rydygier Collegium Medicum in Bydgoszcz, Kurpińskiego 5, 85-950 Bydgoszcz, Poland; m.przybylek@cm.umk.pl; 4 Institute of Advanced Studies, Nicolaus Copernicus University in Toruń, Wileńska 4, 87-100 Toruń, Poland

**Keywords:** sulfamethoxazole, UV/NaOCl treatment, advanced oxidation processes, transformation products, ecotoxicological assessment, in silico screening, environmental fate

## Abstract

UV/NaOCl and UV/H_2_O_2_/NaOCl treatments of sulfamethoxazole (SMX) were compared across several reagent ratios using pseudo-first-order kinetics, LC-DAD/LC-MS screening of transformation products (TPs), multi-trophic bioassays, and quantitative structure–activity relationship (QSAR)-based toxicity and fate screening. Increasing the NaOCl proportion from an SMX:NaOCl molar ratio of 1:1 to 1:10 increased the pseudo-first-order rate constant from 0.215 to 0.818 min^−1^, but faster SMX removal did not correspond to a more favorable post-treatment bioassay profile. The 1:1 and 1:2 UV/NaOCl systems achieved substantial SMX removal within 10 min and gave the lowest responses among the treated systems in the Microtox^®^, Daphtox F^®^, and *Lemna* sp. assays. Higher NaOCl loadings and the mixed-oxidant systems produced stronger post-treatment bioassay responses and larger signals for ECOSAR-prioritized chlorinated aromatics and coupling products. Fate screening further distinguished more persistent, strongly sorbing products from TPs with lower sorption or greater predicted transport potential. The tested reagent ratios therefore revealed a trade-off between removal kinetics and the biological and environmental profile of the resulting mixtures.

## 1. Introduction

Antibiotics are central to infection control and support many medical procedures, including surgery and solid-organ transplantation [1,2]. Their widespread use also contributes to the environmental release of pharmaceutically active residues. Sulfamethoxazole (SMX) is a widely used sulfonamide antibiotic frequently detected in municipal wastewater effluents and surface waters [3,4,5]. Reports of SMX in sediments, groundwater, soil, and drinking-water sources further confirm its broad environmental distribution [5,6,7,8]. This occurrence is largely attributed to incomplete removal during conventional wastewater treatment [5,9]. Consequently, SMX remains detectable in aquatic systems and can affect a range of organisms [10]. A compilation of 5536 measurements in European surface waters reported a median SMX concentration of 52 ng·L^−1^ and a 95th percentile of 286 ng·L^−1^ [11].

Reported ecotoxicological effects in aquatic organisms underscore the need for effective and reliable removal methods applicable to diverse water matrices. Advanced oxidation processes (AOPs) address this need by generating highly reactive oxygen species (ROS), particularly hydroxyl radicals (•OH), which enable efficient degradation of organic pollutants in wastewater. Common AOP configurations include Fenton (Fe^2+^/H_2_O_2_), UV/H_2_O_2_, UV/O_3_, electrochemical oxidation, and TiO_2_ photocatalysis [12,13,14,15]. These systems generate largely non-selective oxidants that can attack a broad range of organic molecules. Accordingly, AOPs such as O_3_/UV, H_2_O_2_/UV, and H_2_O_2_/O_3_/UV can efficiently degrade persistent pharmaceuticals, including ibuprofen and SMX [16].

UV/chlorine requires careful control because it can form halogenated disinfection by-products (DBPs), including trihalomethanes, haloacetic acids, and other emerging species [17,18]. However, DBP formation depends strongly on operating conditions and is not necessarily greater than during conventional chlorination [18].

Operational comparison of UV/chlorine and UV/H_2_O_2_ also depends on energy demand. Under some conditions, UV/chlorine has required substantially less energy for micropollutant removal [19]. Residual H_2_O_2_ from UV/H_2_O_2_ may require quenching before downstream chlorination and can influence DBP formation at that step [20,21].

The enhanced removal efficiency of UV/chlorine is driven by radical reactions. UV activation of free chlorine generates reactive chlorine species such as Cl• and Cl_2_•^−^ together with •OH, expanding the available oxidative pathways relative to systems dominated by •OH [18,19,22]. UV/chlorine can also combine oxidation with disinfection, and NaOCl is a readily available chlorine source in treatment trains where chlorination infrastructure is already present [18,23,24,25]. The operational interest of combining H_2_O_2_ and NaOCl has also been demonstrated in wastewater-reclamation strategies using concurrent oxidant dosing [26].

Free-chlorine transformation of SMX has been characterized in mechanistic chlorination experiments and in distribution-relevant settings [27,28,29]. These studies show that SMX reacts readily with free chlorine, with reaction rates primarily governed by pH and free-chlorine concentration, and that contact with pipe materials can further modulate kinetics in distribution systems. In bromide-containing waters, NaOCl can accelerate SMX loss and shift the product mixture toward brominated species [30]. Bromide-containing chlorination systems have also been associated with more toxic disinfection by-products [30,31]. In aquaculture water, chlorination has also been associated with halogenated by-products, including haloacetic acids [32]. A pilot-scale study of sulfadiazine in a water-distribution system found that differences in transformation pathways coincided with measurable shifts in bacterial community structure. Related studies have examined UV/chlorine and UV or VUV photoprocesses in complex matrices [33,34,35].

Most AOP studies of SMX emphasize parent-compound removal, reaction kinetics, and assignment of selected transformation products [16,34,35,36,37,38,39]. Toxicity is often represented by a single organism or a general endpoint. These aspects have rarely been evaluated together for UV/chlorine treatment of SMX. This study therefore compared UV/NaOCl and UV/H_2_O_2_/NaOCl at several reagent ratios. SMX degradation and time-dependent product profiles were measured alongside bioassay responses in organisms from different trophic levels. The assigned TPs were also screened using persistence, mobility, and toxicity descriptors relevant to the Persistent, Mobile, and Toxic (PMT) concept, which addresses substances that can threaten drinking-water resources through persistence and water-phase mobility [40,41,42]. Experimental profiling and in silico screening were then integrated to prioritize transformation products with PMT-relevant descriptor profiles.

## 2. Results and Discussion

### 2.1. Sulfamethoxazole Degradation Kinetics Under UV-Driven AOPs

**Figure 1:**
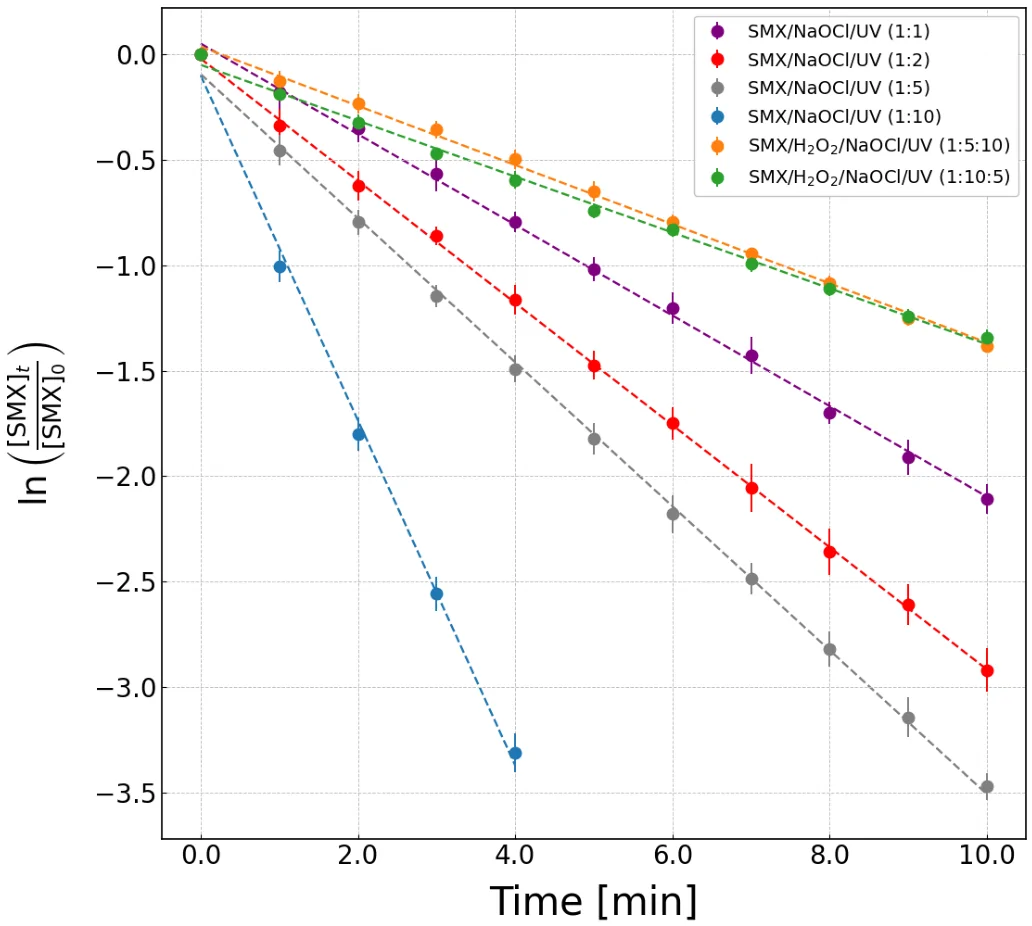
Pseudo-first-order kinetic plots of SMX removal during UV/NaOCl and UV/H_2_O_2_/NaOCl treatment.

Sulfamethoxazole degradation kinetics were evaluated by plotting $ln\frac{{\left[SMX\right]}_{t}}{{\left[SMX\right]}_{0}}$ against time, which gave linear relationships consistent with pseudo-first-order behavior under all tested conditions. The corresponding kinetic plots are presented in Figure 1. A clear hypochlorite-dose response was observed in the UV/NaOCl series, whereas the mixed-oxidant systems showed substantially lower rate constants. Table 1 summarizes the pseudo-first-order parameters obtained here for UV/NaOCl and UV/H_2_O_2_/NaOCl and also includes previously reported results for direct UV and UV/H_2_O_2_ at defined oxidant-to-substrate ratios [10]. Representative literature values for other SMX treatment systems are included for context.

Among the tested systems, UV/NaOCl produced the fastest SMX degradation, with the 1:10 ratio showing a markedly steeper early decline than the other conditions. The pseudo-first-order rate constant (k) increased from 0.215 to 0.818 min^−1^ as the SMX:NaOCl molar ratio increased from 1:1 to 1:10. Although the kinetic profiles clearly separate the 1:10 condition from the slower systems, strict numerical comparison of its k value is limited because the 1:10 analysis was based on a shorter 0–4 min interval and fewer time points. This shorter interval reflected the very rapid decrease in SMX concentration: 96% removal had already been reached by 4 min. By comparison, removal after 10 min was 88%, 95%, and 97% for the 1:1, 1:2, and 1:5 ratios, respectively. The concentration trend indicates that SMX would be near the analytical limit of quantification (LOQ) by 10 min, where quantitative measurements are inherently more uncertain. Importantly, the 0–4 min relationship remained highly linear (R^2^ = 0.996), supporting the quality of the observed early-time trend.

The trends shown in Figure 1 therefore indicate a clear dependence of SMX removal on NaOCl loading within the UV/NaOCl series. The progressively faster decline at higher NaOCl ratios is consistent with the free-chlorine photolysis pathways shown in Equations (1)–(3), which generate Cl•, Cl_2_•^−^, and •OH as reactive species capable of attacking electron-rich sites in SMX.

(1) $$HOCl+h\nu \to HO^{•}+{Cl}^{•}$$

(2) $${OCl}^{-}+h\nu \to {Cl}^{-}+{O•}^{-}$$

(3) $$Cl•+{Cl}^{-}⇌{Cl₂•}^{-}$$

In the mixed UV/H_2_O_2_/NaOCl systems, H_2_O_2_ addition did not increase the degradation rate within the examined ratios. The k values were 0.140 min^−1^ (1:5:10) and 0.133 min^−1^ (1:10:5), corresponding to approximately 75% and 74% parent-SMX removal after 10 min. Thus, the lower rate constants of the mixed-oxidant systems were also reflected in lower parent-compound removal at the common 10 min endpoint. UV photolysis of H_2_O_2_ can generate additional •OH, while H_2_O_2_ can also consume active chlorine and reactive chlorine species, thereby reducing their availability for the chlorine-radical pathway described in Equations (4) and (5). Similar neutral or slightly negative effects of adding H_2_O_2_ have been reported for other targets during UV/chlorine treatment, including ibuprofen, gemfibrozil, naproxen [50], and methylene blue at low peroxide dose [51].

(4) HOCl+H2O2→H3O++Cl−+O21

(5) $${Cl}^{•}+H_{2}O_{2}+H_{2}O\to H_{3}O^{+}+{Cl}^{-}+{HO}_{2}^{•}$$

The closest literature comparison is our earlier study, performed with the same initial SMX concentration, pH, temperature, and 150 W medium-pressure lamp [10]. The present study extends this experimental series to chlorine-containing systems, complementing the previously reported direct UV, UV/H_2_O_2_, and photo-Fenton conditions. Under those conditions, UV alone gave k = 0.106 min^−1^, UV/H_2_O_2_ gave k = 0.120–0.136 min^−1^, and photo-Fenton at pH 5 gave k = 0.186–0.709 min^−1^. Thus, the UV/NaOCl variants at 1:5 and 1:10 (k = 0.342 and 0.818 min^−1^) were approximately 3.2 and 7.7 times faster than UV alone, whereas changing the H_2_O_2_ ratio from 1:1 to 1:30 changed k by less than 15%. Under these closely matched conditions, the dose response was therefore substantially stronger for free chlorine than for H_2_O_2_. Across the broader literature compilation in Table 1, k spans more than two orders of magnitude because irradiation, oxidant dose, pH, and matrix differ markedly. Matrix effects alone can be large: at the same SMX:NaOCl ratio of 1:10, reported k values were 0.08, 1.69, and 3.44 min^−1^ in fresh water, marine culture water, and marine water, respectively [30]. Cross-study rate constants should therefore be interpreted in the context of the experimental matrix rather than ranked as directly equivalent treatment efficiencies.

It is worth noting that the controlled model matrix, while useful for isolating the effect of reagent ratio, limits direct transferability of the results to natural waters. Its defined composition allowed SMX removal, TP formation, and mixture toxicity to be compared without concurrent changes in background water chemistry. In real waters, however, dissolved organic matter and inorganic constituents can alter oxidant demand, degradation kinetics, and product distribution [30]. The present conclusions therefore apply to the tested model conditions. Further validation in representative real-water matrices is needed to determine how matrix composition influences the observed treatment performance, including degradation behavior, transformation-product profiles, and post-treatment biological responses.

### 2.2. Transformation Products

**Figure 2:**
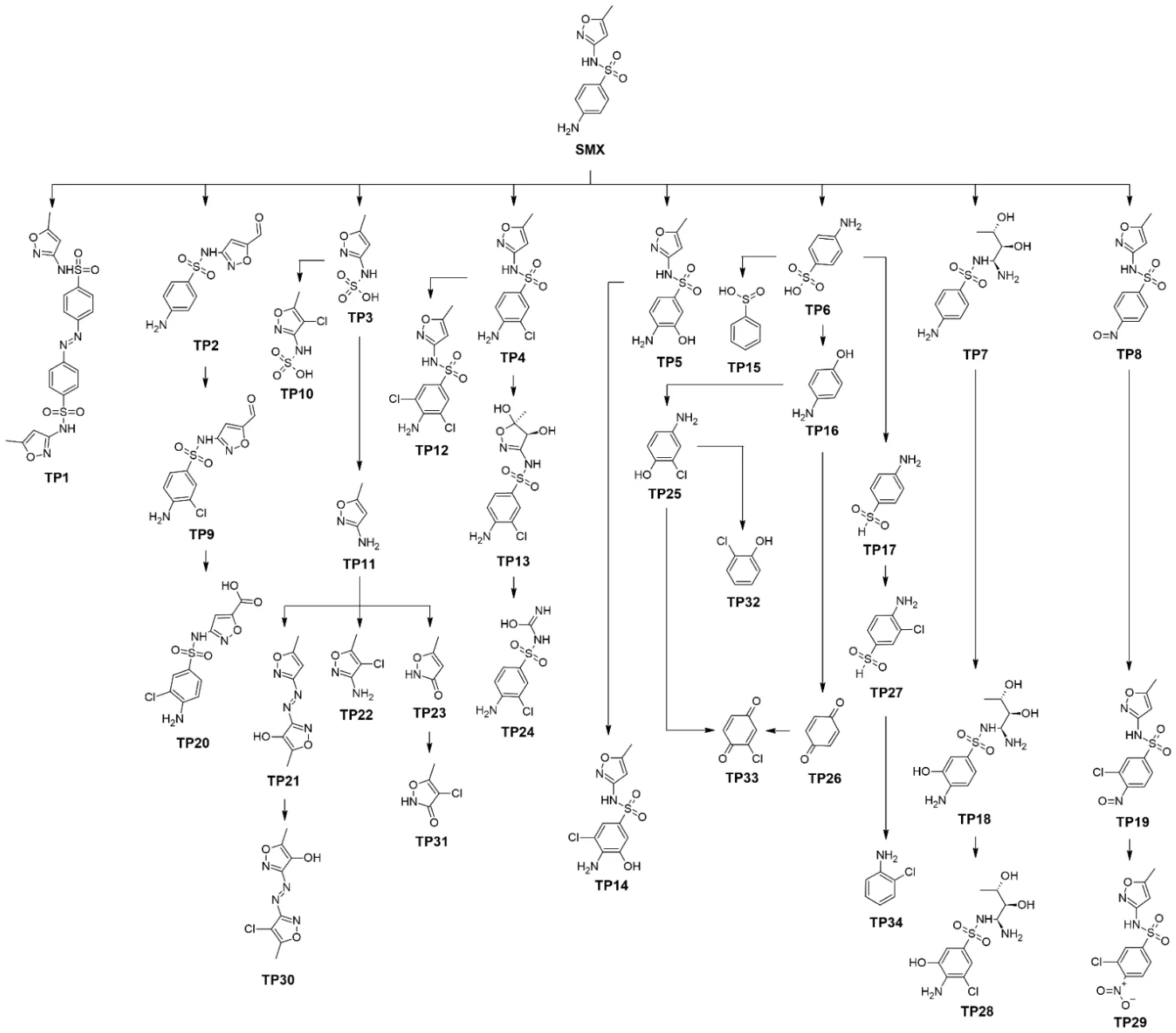
LC-MS-assigned transformation products and plausible transformation relationships proposed for SMX under UV-induced chlorinating and oxidative conditions.

The transformation products (TPs) assigned for sulfamethoxazole (SMX) are summarized in Figure 2. A comprehensive list of proposed structures (SMILES), diagnostic ions (*m*/*z*), and the treatment conditions in which each product was detected is provided in the Supplementary Materials (Supplementary_Data.xlsx, sheet “Detected compounds”). TP1 to TP34 included chlorinated derivatives, oxygenated species including hydroxylated products, ring-modified or ring-opened compounds, coupling products, and structurally simpler aromatic products, including chlorinated phenols and chloroanilines. The assigned TPs indicate transformations involving the sulfonamide linkage, the aniline ring, and the isoxazole moiety. The assignment of TP6 is consistent with cleavage of the S–N bond. Chlorination of the aromatic ring was represented by mono- and dichloro congeners, including TP4 and TP12. This pattern is consistent with electrophilic aromatic substitution reported for chlorination and UV/chlorine treatment [27,28,29]. Azo-linked coupling products such as TP1, TP21, and TP30 were also assigned and are typically attributed to radical coupling of aromatic intermediates formed during radical-driven treatment [48,52,53]. Hydroxylated products were assigned under UV/NaOCl conditions, including TP5 and TP16. TP16 was also present under UV/H_2_O_2_/NaOCl, indicating that hydroxylation can also occur under the mixed-oxidant conditions.

In addition to reactions affecting the sulfonamide linkage and the aniline ring, several TPs indicate direct modification of the isoxazole moiety under the applied conditions. TP13 is consistent with hydration accompanied by partial saturation of the isoxazole ring. Isoxazole-ring opening was represented by hydroxylated sulfonamide derivatives TP7 and TP18, together with the chlorinated analog TP28 [52,54,55]. Further oxidation beyond simple substitution is suggested by quinone-type products TP26 and TP33. Phenolic products and aromatic amines, including TP25, TP32, and TP34, were also assigned. Nitroso and nitro products were assigned under NaOCl/UV conditions, including TP8, TP19, and TP29, but they represented only a small part of the assigned TP set.

### 2.3. Ecotoxicity of Reaction Mixtures

**Figure 3:**
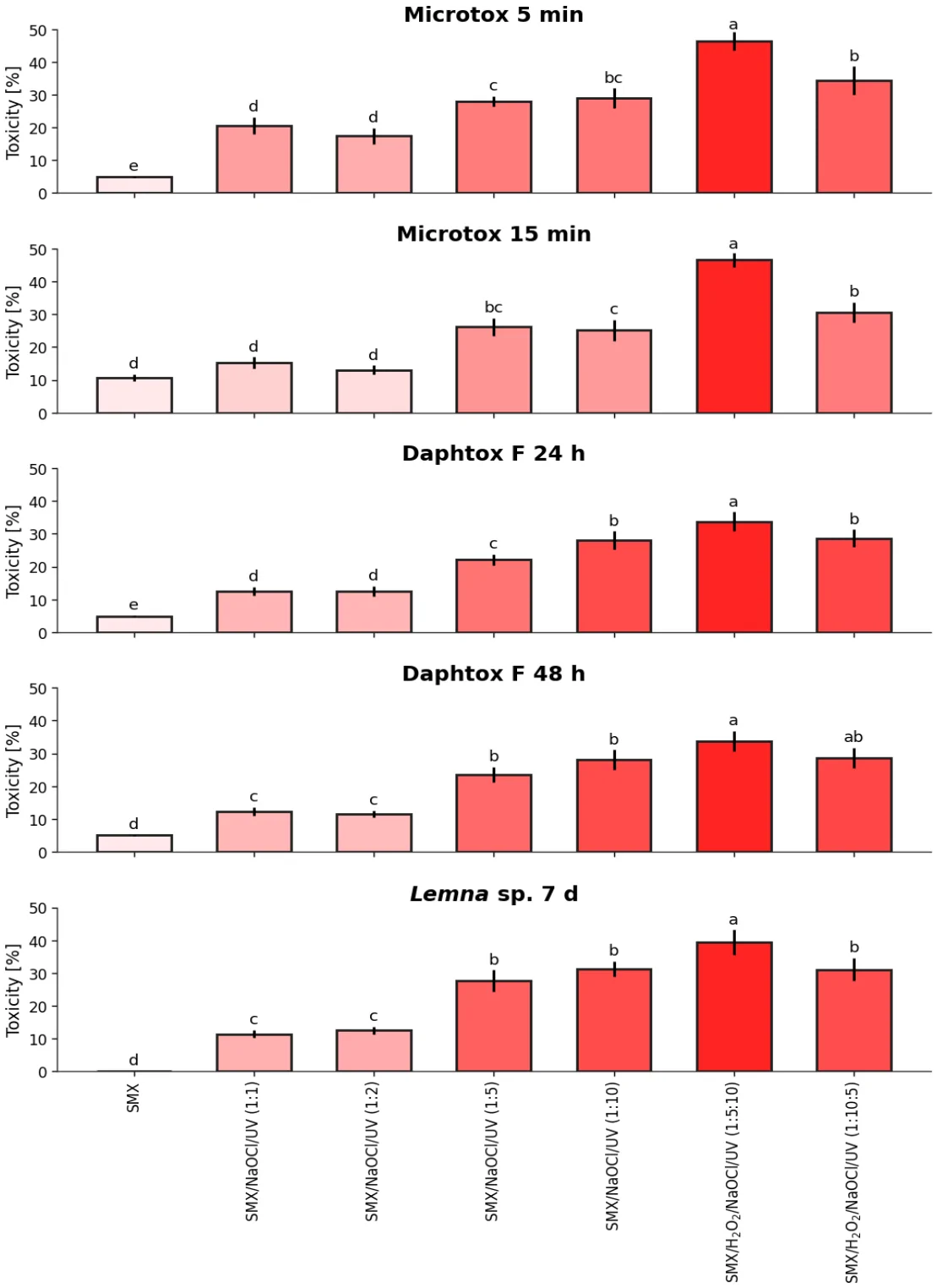
Post-treatment ecotoxicity of reaction mixtures collected after 10 min of UV-based treatment, assessed by Microtox^®^ (5 and 15 min), Daphtox F^®^ (24 and 48 h), and *Lemna* sp. growth inhibition (7 d). Untreated SMX is shown for comparison. Error bars indicate SD (*n* = 4). Letters show Tukey’s HSD groupings (*p* < 0.05), calculated separately for each endpoint. Shared letters indicate no significant differences.

Figure 3 summarizes the post-treatment responses across the three bioassays: *Aliivibrio fischeri* luminescence inhibition [56], *Daphnia magna* immobilization [57], and *Lemna* sp. growth inhibition [58]. Across the three endpoints, responses generally increased from the low-NaOCl UV/NaOCl systems toward conditions with higher NaOCl loading or mixed oxidants.

At both Microtox^®^ exposure times, the two low-hypochlorite systems gave the weakest responses among the treated samples. After 5 min, luminescence inhibition in *Aliivibrio fischeri* was 20.5% at 1:1 and 17.4% at 1:2, compared with 46.3% for SMX/H_2_O_2_/NaOCl/UV (1:5:10). A similar ordering was observed at 15 min, with the mixed 1:5:10 system remaining the most toxic treatment. The agreement between the two exposure times is consistent with a treatment-dependent contribution of the product mixture after substantial SMX removal. Previous studies have similarly reported an initial decrease in toxicity followed by a renewed increase as transformation products accumulated [37,55,59].

The Daphtox F^®^ assay showed a consistent pattern across treatments. Responses after 24 and 48 h were broadly similar, with no systematic increase over time. At 48 h, *Daphnia magna* immobilization was 12.4% and 11.6% for the 1:1 and 1:2 SMX/NaOCl/UV systems, respectively. By comparison, immobilization reached 33.7% for SMX/H_2_O_2_/NaOCl/UV (1:5:10).

Our earlier work under closely matched conditions provides a useful UV-only reference for these results. Direct UV treatment at pH 5 did not achieve detoxification, and the post-UV mixture produced stronger bioassay responses than untreated SMX [10]. Similar observations have been reported for other photochemical and oxidative treatments of SMX. Direct UV photolysis can increase mixture toxicity as photoproducts accumulate [60]. Transient and organism-dependent changes in toxicity have also been observed during SMX photodegradation [37]. During ozonation, rapid removal of the parent compound likewise did not result in immediate detoxification, as toxicity changed while reaction intermediates formed and subsequently decayed [54]. Taken together, these findings show that removal of the parent compound alone does not adequately describe the biological quality of the treated mixture.

In the 7 d *Lemna* sp. assay, growth inhibition within the UV/NaOCl series increased with NaOCl loading, with the lowest responses observed for the 1:1 and 1:2 systems. The mixed 1:10:5 system gave a response similar to the high-NaOCl UV/NaOCl condition, whereas 1:5:10 produced the strongest inhibition among the treatments. The similar ordering in the Microtox^®^, Daphtox F^®^, and *Lemna* sp. assays indicates that the treatment-dependent effect was observed across all three test organisms.

**Figure 4:**
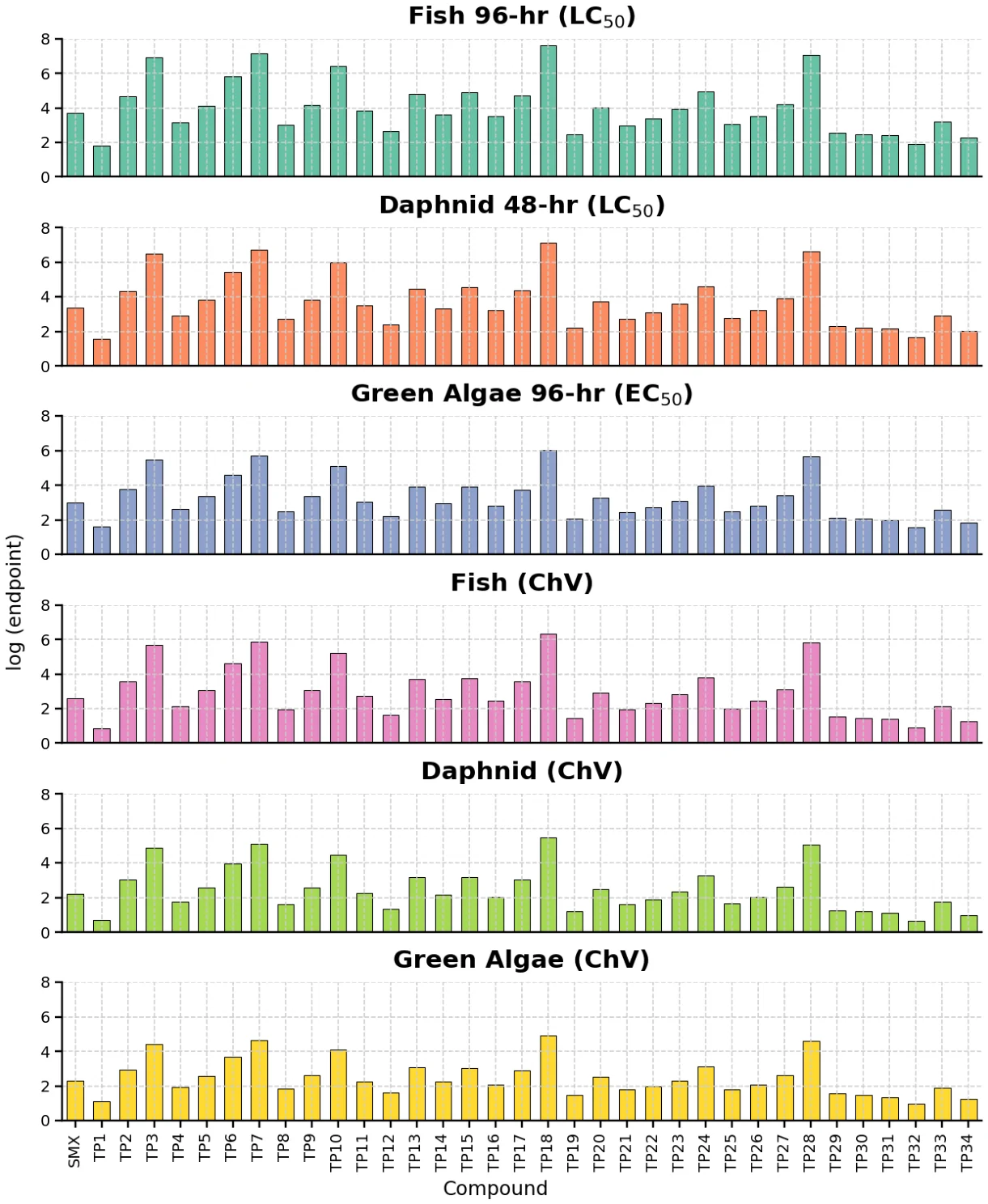
ECOSAR-predicted aquatic toxicity of sulfamethoxazole (SMX) and the assigned TPs across fish, daphnid, and green algae endpoints. Median lethal concentration (LC_50_), median effect concentration (EC_50_), and chronic value (ChV) estimates [mg·L^−1^] are provided in Supplementary_Data.xlsx, sheet “EPISuite”.

According to the ECOSAR predictions shown in Figure 4 and Supplementary_Data.xlsx (sheet “EPISuite”), the lowest predicted effect concentrations across fish, daphnid, and green algae endpoints were associated mainly with low-molecular-weight chlorinated aromatics, particularly chlorophenol (TP32) and chloroaniline (TP34). The azo-coupling products TP1 and TP30 also ranked highly, as did the chlorinated products TP31 and TP19. TP31 was assigned as a chlorinated isoxazolinone, whereas TP19 was a nitroso-bearing aromatic sulfonamide. Most prioritized compounds therefore belonged to product families formed under UV/NaOCl, especially chlorinated aromatics and coupling products. Conditions with greater hypochlorite excess also produced stronger Daphtox F^®^ immobilization and *Lemna* growth inhibition.

**Figure 5:**
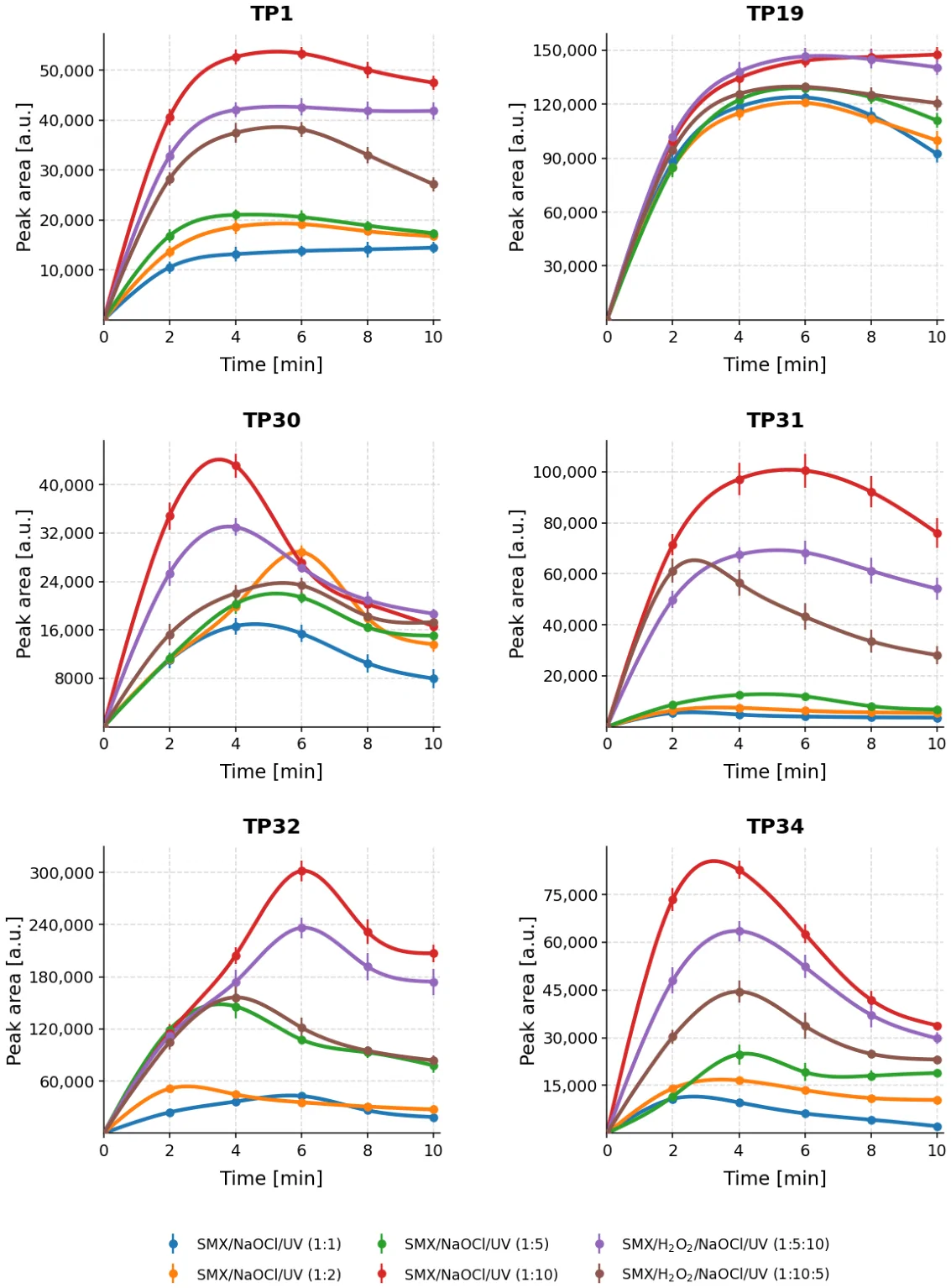
Time profiles of six transformation products prioritized by ECOSAR (TP1, TP19, TP30, TP31, TP32, and TP34) in UV/NaOCl and UV/H_2_O_2_/NaOCl systems. Profiles are shown as LC-MS peak-area signals [a.u.].

To compare the bioassay results with the behavior of individual transformation products, Figure 5 shows the time profiles of the six products with the lowest ECOSAR-predicted effect concentrations: the coupling products TP1 and TP30 and the chlorinated products TP19, TP31, TP32, and TP34. Selection was rank-based rather than defined by an arbitrary numerical cutoff. These six compounds occupied ranks 1–6 for every ECOSAR endpoint evaluated, whereas TP29 was ranked seventh across all six endpoints (Supplementary_Data.xlsx, sheet “EPISuite”). The profiles in Figure 5 are chromatographic peak-area signals and therefore support relative temporal and between-treatment comparisons rather than absolute TP concentrations. TP30 differed from the other selected products because its signal reached a maximum earlier and subsequently declined under several conditions. Treatment-dependent differences among the six products are discussed below. This approach therefore prioritized compounds showing consistently high predicted toxicity across the complete ECOSAR endpoint set.

All six monitored TP signals were detectable at 2 min and remained detectable at the 10 min sampling point. TP30 showed the clearest decrease after reaching an early maximum under several treatment conditions. TP1, TP31, TP32, and TP34 also increased initially and then either declined or approached a plateau. TP19 followed a different pattern, generally reaching its highest signal between 6 and 10 min. Under the highest NaOCl loading, its signal was still increasing at 10 min.

These profiles indicate that the transformation-product composition was still changing during the treatment period. At the 10 min endpoint used for the bioassays, all six prioritized TP signals remained detectable, and TP19 was still increasing under the highest NaOCl loading. While the signal of an individual product may decrease, this alone does not indicate that the overall toxicity of the mixture is also decreasing. The treated samples still contained multiple transformation products with different time profiles at the bioassay endpoint.

A comparable evolution of toxicity has been reported during treatment with free chlorine in the absence of UV. Toxicity increased during early chlorination as reactive chlorinated intermediates accumulated and declined only after several hours of further degradation [27]. Products formed from SMX, including low-molecular-weight heteroaromatic fragments such as 3-amino-5-methylisoxazole, have shown higher toxicity toward *Daphnia magna* and photosynthetic primary producers than SMX, although weaker effects on fish have also been reported [61]. Chronic exposure to SMX itself can impair feeding, locomotion, lipid metabolism, and reproduction in *Daphnia magna* at concentrations relevant to the environment, including effects associated with acetylcholinesterase and lipase inhibition [62]. In the present study, systems with higher NaOCl concentrations likewise retained large signals from transformation products at 10 min and produced stronger Microtox^®^ and *Daphnia* responses.

Treatment-level comparison showed a consistent separation between the lower- and higher-oxidant conditions. At 1:1 and 1:2, product signals remained comparatively low and often leveled off or declined after 4–6 min. The largest signals were generally obtained for the 1:10 UV/NaOCl condition, while the H_2_O_2_-containing mixtures also showed elevated signals for several prioritized products. The 1:5 UV/NaOCl condition was intermediate and product-dependent. Overall, the treatment ordering in Figure 5 broadly paralleled that in Figure 3: conditions producing stronger prioritized-product signals also produced stronger bioassay responses, whereas the two low-hypochlorite systems gave the weakest responses in both comparisons.

Within the tested ratios, adding H_2_O_2_ neither accelerated SMX removal nor reduced the 10 min signals of the prioritized halogenated and partially oxidized products. The H_2_O_2_-containing mixtures also produced stronger post-treatment bioassay responses than the 1:1 and 1:2 UV/NaOCl systems (Figure 3). Thus, the extent of SMX removal alone did not reflect the biological response after treatment.

**Figure 6:**
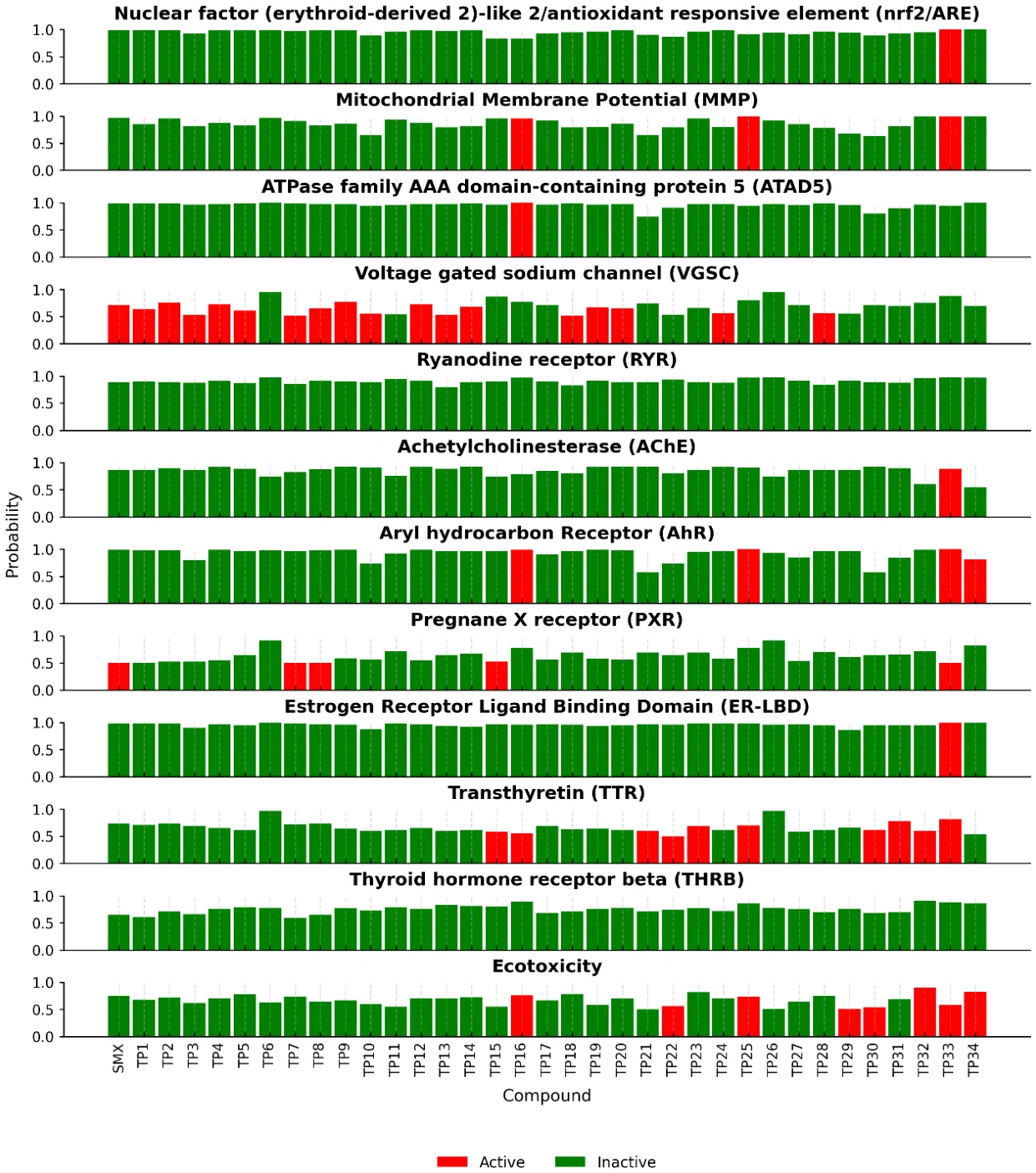
Selected ProTox predictions for toxicity endpoints and molecular targets for SMX and the assigned transformation products. The complete ProTox output is provided in Supplementary_Data.xlsx, sheet “ProTox”.

ProTox was then used to complement the ECOSAR assessment by examining additional toxicity endpoints and molecular targets for SMX and the assigned transformation products (Figure 6). The complete output is provided in Supplementary_Data.xlsx (sheet “ProTox”).

Endocrine-related ProTox predictions were limited rather than widespread across the assigned TPs (Figure 6). TP33 was the only product predicted to be active in the estrogen receptor ligand-binding domain (ER-LBD) endpoint, with a probability of 1.00. Transthyretin (TTR)-binding alerts were predicted for TP15, TP16, TP21, TP22, TP23, TP25, TP30, TP31, TP32, and TP33, with probabilities of 0.50 to 0.82. These alerts were concentrated in selected structural groups, including phenolic and related aromatic products, small isoxazole or isoxazolinone derivatives, and azo-coupling products; several of these compounds were chlorinated. This limited pattern is consistent with previous experimental studies reporting no estrogenic activity of SMX and no detectable estrogenicity after oxidative treatment of SMX-spiked wastewater [63,64]. SMX is also reported as inactive in the Tox21 ERα binding dataset used for predictive modeling [65].

ProTox classified eight of the 35 screened compounds as active for the ecotoxicity endpoint. TP32 (0.91) and TP34 (0.83) had the highest predicted probabilities, followed by TP16 (0.76) and TP25 (0.74). Alerts were also obtained for nuclear factor erythroid 2-related factor 2/antioxidant response element (Nrf2/ARE), a pathway involved in cellular redox regulation, and mitochondrial membrane potential (MMP). Perturbations of redox regulation and MMP are associated with oxidative stress and impaired cellular function during short exposures [66,67,68,69]. These outputs help prioritize selected TPs for further testing, but they do not by themselves explain the Microtox^®^ or *Lemna* sp. responses.

Neurotoxicity-related predictions were likewise heterogeneous. Voltage-gated sodium channel (VGSC) activity was predicted for the largest number of compounds, whereas the ryanodine receptor (RYR) endpoint was inactive for all screened structures. Acetylcholinesterase (AChE) activity was predicted only for the chlorinated benzoquinone TP33. This AChE alert is consistent with reported inhibition by 1,4-benzoquinone derivatives and with reduced AChE activity after halobenzoquinone exposure in vivo [70,71]. Altered AChE activity has also been associated with toxic and behavioral effects in aquatic organisms, including zebrafish and *Daphnia magna* [72,73,74].

VGSC activity was predicted for SMX and many TPs, including several chlorinated products. However, the corresponding ECOSAR estimates for *Daphnia* covered a broad range. The VGSC alert did not track consistently with predicted daphnid toxicity and did not distinguish the TPs from the parent compound. Aryl hydrocarbon receptor (AhR) and pregnane X receptor (PXR) activity was also predicted for selected compounds.

Among the profiled transformation products, TP33 showed the broadest ProTox activity profile, with 14 active outputs, followed by TP25 with 10. For TP25, predicted probabilities for both MMP and AhR reached 1.00. Predicted ATPase family AAA domain-containing protein 5 (ATAD5) activity was much more selective and occurred only for TP16 (4-aminophenol). This redox-active aromatic amine was also predicted to be active for MMP and AhR and has caused DNA strand breaks and chromosomal alterations in cell-based assays [75,76].

Across the TP set, no single shared toxicological mechanism emerged. ProTox highlighted TP33 and TP25 as compounds with the broadest predicted activity profiles, while other TPs showed more selective, endpoint-specific alerts. This screening is useful for prioritizing compounds and endpoints for targeted experimental testing, but it cannot identify which individual compounds caused the measured mixture responses.

### 2.4. Environmental Fate and Mobility of Transformation Products

Environmental fate calculations were used to compare SMX and the assigned transformation products in terms of persistence, long-range transport, sorption, water solubility, and bioaccumulation. EPI Suite estimates were combined with overall persistence (P_OV_), characteristic travel distance (CTD), and transfer efficiency (TE) values from the OECD persistence and long-range transport potential (LRTP) screening tool. The fate descriptors provided the persistence and mobility component of the screening, while toxicity was assessed from the bioassays and in silico results described above [40,41,42,77,78]. Sorption and solubility are reported below as the organic carbon-normalized sorption coefficient (K_OC_) and water solubility (S), respectively.

**Figure 7:**
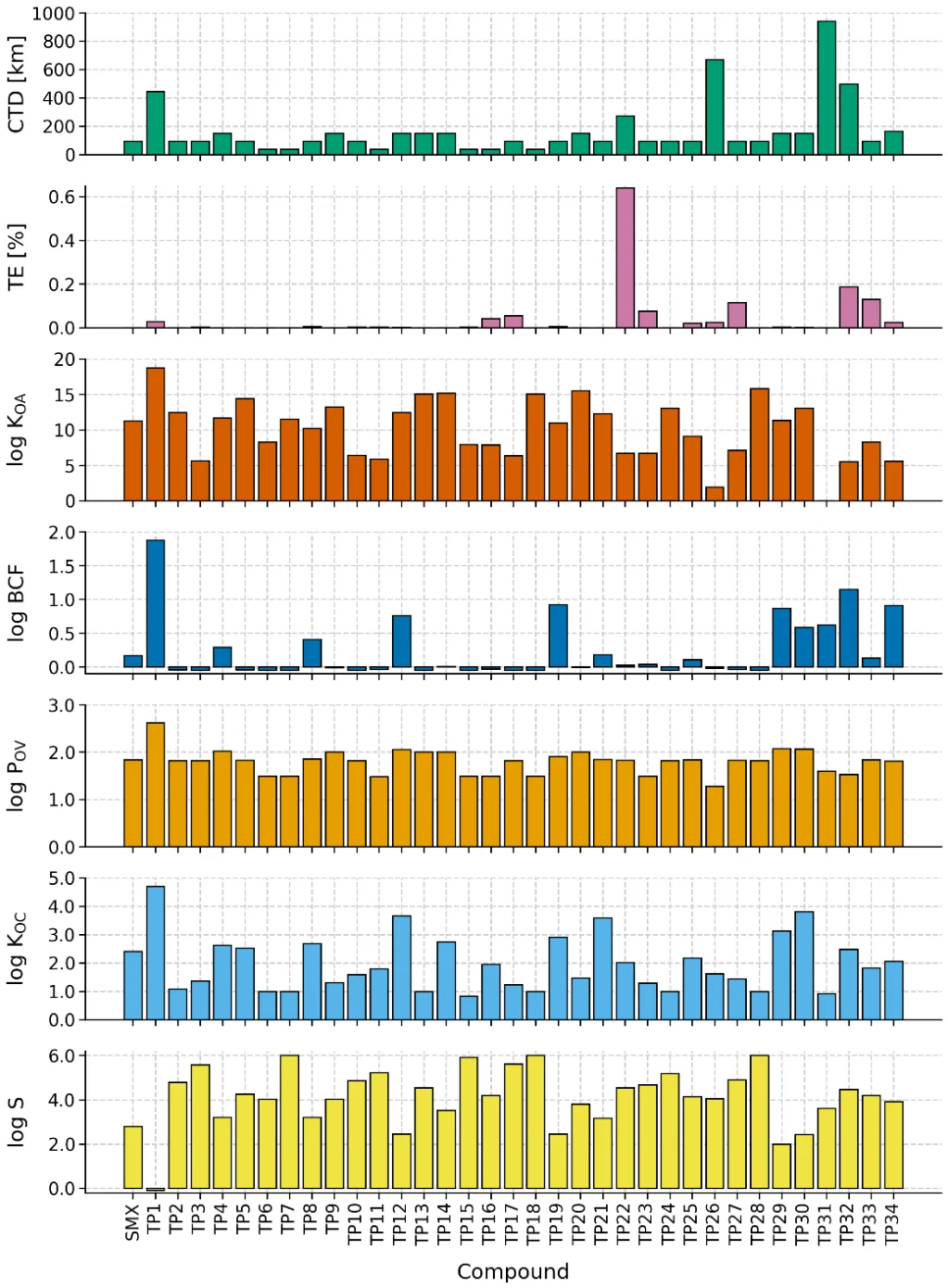
Predicted environmental fate descriptors for sulfamethoxazole (SMX) and its TPs. P_OV_, CTD, and TE were obtained with the OECD P_OV_ and LRTP screening tool. The octanol–air partition coefficient (K_OA_), bioconcentration factor (BCF), organic carbon-normalized sorption coefficient (K_OC_), and water solubility (S, mg·L^−1^) were estimated with EPI Suite.

Figure 7 shows that the assigned TPs span a broad range of persistence, transport, sorption, and solubility. Log P_OV_ ranged from 1.28 to 2.62, CTD from 37.37 to 940.44 km, log K_OC_ from 0.84 to 4.71, and log S from −0.09 to 6.00. Complete numerical values for SMX and all assigned TPs are provided in Supplementary_Data.xlsx (sheet “EPISuite”).

Comparison with SMX shows how transformation shifted the predicted fate properties. SMX combined moderate predicted persistence (log P_OV_ = 1.84) and limited long-range transport (CTD = 93.35 km, TE = 0.0004%) with high water solubility (log S = 2.79), low bioaccumulation potential (log BCF = 0.17), and moderate sorption (log K_OC_ = 2.41). This combination is consistent with appreciable aqueous mobility, although matrix properties can substantially modify transport. Soil-column studies describe SMX as moderately to highly mobile, with pH and organic carbon content among the main controlling factors [79,80]. Lower uptake in zebrafish in the presence of sediment has likewise been linked to reduced aqueous availability after sorption [81].

The more persistent TPs differed markedly in their mobility-related properties. TP1 had the highest log P_OV_ (2.62) and relatively high long-range transport indicators (CTD = 445.53 km, TE = 0.0281%), but it also had the highest log K_OC_ (4.71) and a high log K_OA_ (18.75), indicating strong partitioning toward organic phases. TP29 and TP30 had similar predicted persistence (log P_OV_ = 2.07 for both), but their sorption-related properties differed: log K_OC_ was 3.14 for TP29 and 3.81 for TP30, while TP30 also had relatively low solubility (log S = 2.43). Thus, similar predicted persistence did not imply similar sorption or solubility [40,41].

Several lower-molecular-weight TPs showed a different profile, combining high solubility with low sorption and negligible predicted bioaccumulation. TP6 and TP16 had log S values of 4.03 and 4.20 and log BCF values below 0. Their log K_OC_ values were 1.00 and 1.96, respectively. TP11 was even more soluble (log S = 5.21), with log BCF = −0.04 and log K_OC_ = 1.79. TP20 was more persistent (log P_OV_ = 2.00) but also combined high solubility (log S = 3.80) with low sorption (log K_OC_ = 1.48). Thus, a relatively persistent TP could still have descriptors compatible with greater water-phase availability [41,77].

The fate analysis also complemented the ECOSAR prioritization described in Section 2.3 by showing how the selected products differed in predicted transport and sorption. Among the chlorinated TPs followed in Figure 5, TP31 had a particularly high predicted CTD (940.44 km) together with low sorption (log K_OC_ = 0.93) and high solubility (log S = 3.61). TP32 combined CTD = 496.10 km with high solubility (log S = 4.45), while TP34 showed CTD = 164.31 km, log S = 3.91, and log K_OC_ = 2.06. TP19 was more strongly sorbing (log K_OC_ = 2.91) and less soluble (log S = 2.44). All four were included in the ECOSAR prioritization, but their predicted transport and sorption properties differed markedly.

TP26 combined relatively low predicted persistence (log P_OV_ = 1.28) with a high CTD of 672.15 km and TE of 0.0243%. Its high solubility (log S = 4.05), negligible bioaccumulation (log BCF = −0.02), and low sorption (log K_OC_ = 1.62) are consistent with substantial predicted transport and water-phase availability. TP26 was detected under UV/NaOCl but not under UV/H_2_O_2_/NaOCl.

Previous work has detected chlorinated SMX-derived by-products across successive stages of wastewater treatment [82]. In the present study, several chlorinated TPs were also prioritized by ECOSAR and showed larger LC-MS signals under conditions that produced stronger bioassay responses.

## 3. Materials and Methods

The experimental design followed a strategy previously used for pharmaceuticals and personal care products (PPCPs) [10,83,84,85]. The treatment conditions were compared using the same set of chemical, biological, and fate-related endpoints: SMX removal, LC-MS product profiles, standardized bioassay responses, and QSAR-based descriptors.

### 3.1. Chemicals and Reagents

Sulfamethoxazole (SMX, ≥98%) was used as the target compound for photodegradation and was purchased from Merck (Darmstadt, Germany). The solvents, HPLC-grade water, acetonitrile (≥99.93%), and formic acid (≥98%), as well as the buffer components citric acid monohydrate (≥99.0%) and trisodium citrate dihydrate (≥99.0%), were also obtained from Merck. Hydrogen peroxide (H_2_O_2_, 30% *w*/*v*) and sodium hypochlorite (NaOCl, 10% active chlorine), used in the reaction mixtures, were purchased from Avantor (Gliwice, Poland).

### 3.2. Experimental Procedure for SMX Degradation

All chemicals and reagents were used as received without further purification, and all treatment experiments were conducted under UV irradiation. All reactions were continued for 10 min, and reagent concentrations are summarized in Table 2. For kinetic analysis, the pseudo-first-order fit for the 1:10 condition used the 0–4 min interval, whereas the other tested conditions used the full 0–10 min profiles. The initial pH of each reaction mixture was adjusted to 5.0 with 0.1 M citrate buffer before irradiation. Across the tested conditions, post-treatment pH remained within a narrow range of 4.8–5.1. Experiments were carried out in a water-cooled photoreactor (Heraeus, Hanau, Germany) with a 150 W medium-pressure mercury lamp housed in a quartz immersion tube. Temperature was maintained at 20 ± 1 °C and the solution was continuously stirred magnetically. Each experiment was performed at a total reaction volume of 700 mL.

### 3.3. Chromatographic Analysis

Quantification of SMX and screening of transformation products were performed using a UFLC XR liquid chromatograph (Shimadzu, Kyoto, Japan) equipped with a diode-array detector (DAD, Nexera X2 SPD-M30A; Shimadzu, Kyoto, Japan) and an LC-MS 2020 mass spectrometer (Shimadzu, Kyoto, Japan). Separation was achieved on a Kinetex C18 column (3.0 × 100 mm, 2.6 μm; Phenomenex, Torrance, CA, USA), using 0.1% (*v*/*v*) formic acid in water as eluent A and acetonitrile as eluent B. The gradient program had a total run time of 8.0 min. The mobile phase was 80% A/20% B from 0.00 to 1.80 min and then changed linearly to 20% A/80% B from 1.80 to 2.50 min. It was held at 20% A/80% B from 2.50 to 5.00 min, returned linearly to 80% A/20% B from 5.00 to 6.50 min, and re-equilibrated at 80% A/20% B from 6.50 to 8.00 min. The operating conditions were a flow rate of 0.40 mL·min^−1^, a column temperature of 30 °C, and an injection volume of 5 μL. SMX concentration was determined using the DAD signal at 268 nm. The calibration response was linear over the tested range (R^2^ = 0.9995). The resulting limit of detection (LOD) and LOQ were 10.2 and 30.6 ng·mL^−1^, respectively. These values were determined from spiked solutions using signal-to-noise criteria, with LOD defined as the lowest level providing S/N ≥ 3 and LOQ as the lowest level providing S/N ≥ 10.

Transformation products were screened by LC-MS under positive electrospray ionization. For SMX, the *m*/*z* 254 ion was monitored in selected-ion monitoring (SIM) mode, while full-scan acquisition over *m*/*z* 50–700 was used to screen additional LC-MS features. The mass spectrometer was operated with nebulizing nitrogen gas at 1.5 L·min^−1^ and drying nitrogen gas at 15 L·min^−1^. The ion-source thermal settings were 250 °C for the desolvation line and 400 °C for the heating block. The probe voltage was 4000 V, and the Qarray DC voltage was 70 V. Chromatographic data were processed in LabSolutions software, version 5.60 (Shimadzu, Kyoto, Japan). Smoothing was performed using the Savitzky–Golay filter. Noise and baseline drift were assessed during data processing. Structural assignments of degradation products were based on diagnostic MS data reported in the literature [30,48,52,54,55,60,82,86,87,88,89,90,91,92,93]. For chlorinated products, chlorine isotope-pattern inspection was also used. The selected-ion chromatogram for SMX and extracted-ion chromatograms for the assigned transformation-product signals are provided in Supplementary_Chromatograms.

### 3.4. Ecotoxicity Assessment

Ecotoxicity was assessed using three standardized aquatic bioassays. These included the Microtox^®^ acute toxicity test with *Aliivibrio fischeri* at 5 and 15 min, the Daphtox F^®^ acute immobilization test with *Daphnia magna* (ISO 6341:2012 [94] and OECD Test No. 202 [95]) at 24 and 48 h, and the *Lemna* sp. Growth Inhibition Test (OECD Test No. 221 [96]) over 7 d. All three assays are established methods used across many matrices and contaminant classes [10,83,84,85,97,98,99,100].

The bioassays were performed on aliquots collected after 10 min of treatment for each condition. This common endpoint enabled direct comparison of the reagent ratios at the same treatment duration. The aliquots were diluted 1:100 (*v*/*v*) with water and tested using the procedures described below. For comparison, the toxicity of an untreated SMX solution containing no oxidants and without exposure to UV irradiation was also determined following the same 1:100 (*v*/*v*) dilution. Control samples containing all components except SMX were processed under identical conditions. For each system, toxicity was calculated as the response of the SMX-containing sample after subtracting the response measured for the corresponding control.

Microtox measurements were performed using the MicrotoxOmni Screening Test on a Model 500 Analyzer (Modern Water Inc., New Castle, DE, USA), with bioluminescence inhibition recorded at 5 and 15 min. The Daphtox F^®^ procedure (TIGRET Sp. z o.o., Warsaw, Poland) followed ISO 6341 and OECD 202. Immobilization was recorded at 24 h and 48 h. In the *Lemna* sp. assay, in-house cultures were exposed for 7 days. The assay was carried out at 25 °C under continuous illumination (6000 lx) to evaluate growth inhibition.

Each condition was tested in four replicates (*n* = 4). Normality and homogeneity of variance were evaluated using the Shapiro–Wilk, Levene, and Brown–Forsythe tests. Group differences were analyzed by one-way ANOVA followed by Tukey’s HSD post hoc test. All statistical analyses were performed using STATISTICA v13 (Dell Inc., Round Rock, TX, USA) [101].

### 3.5. Prediction of Biological Effects and Toxicological Potential

To complement the experimental ecotoxicity assessment, the potential toxicological effects of SMX and its TPs were evaluated using in silico screening tools. Aquatic hazard was predicted with the ECOSAR module included in the Estimation Programs Interface Suite (EPI Suite, version 4.11) [102], developed by the U.S. Environmental Protection Agency (Washington, DC, USA). ECOSAR uses chemical class-specific QSAR relationships and SMILES-encoded structures to estimate EC_50_, LC_50_, and chronic toxicity values (ChV). Predictions were generated for three standard aquatic organism groups: fish, *Daphnia magna*, and green algae.

A broader toxicological profile was obtained using the ProTox 3.0 platform (https://tox.charite.de/protox3/; accessed on 27 July 2026) [103,104,105]. This web-based tool applies chemical similarity analysis, fragment-based structural descriptors, and machine-learning models to predict toxicological endpoints. The outputs used in this study included hepatotoxicity, neurotoxicity, mutagenicity, ecotoxicity, carcinogenicity, and immunotoxicity. In addition, potential activation of nuclear receptor pathways was evaluated, with emphasis on endocrine-related signaling [106].

### 3.6. Prediction of Physicochemical Properties and Environmental Fate

Physicochemical properties and environmental fate parameters of SMX and the TPs were estimated using EPI Suite. The analysis relied primarily on model-estimated values and, where available, was supplemented with experimental entries from the integrated database. Partitioning behavior was characterized using descriptors relevant to environmental distribution, volatility, and mobility. Sorption potential was estimated as the organic carbon-normalized sorption coefficient (K_OC_, reported as logK_OC_) using the KOCWIN module and the molecular connectivity index (MCI) method. Water solubility (S) was estimated with the WSKOWWIN module. Air-related partitioning was described using the air–water (logK_AW_) and octanol–air (logK_OA_) partition coefficients.

Degradation half-lives in air, water, and soil were estimated using the BIOWIN and HYDROWIN modules and were subsequently integrated with selected partitioning descriptors as inputs for the OECD P_OV_ and LRTP screening tool [107,108]. These outputs provided preliminary estimates of overall persistence (P_OV_) and long-range transport potential (LRTP). The resulting values were used to compare persistence and transport-related properties across the assigned TPs.

## 4. Conclusions

This study showed that faster SMX removal during UV/chlorine treatment did not translate into lower post-treatment bioassay responses. Increasing the NaOCl proportion across SMX:NaOCl molar ratios from 1:1 to 1:10 raised the pseudo-first-order rate constant from 0.215 to 0.818 min^−1^. Despite this faster removal, the 1:1 and 1:2 systems produced the weakest responses among the treated samples in all three bioassays while still achieving substantial SMX removal within 10 min. Higher NaOCl loadings and the mixed UV/H_2_O_2_/NaOCl systems produced stronger bioassay responses and larger signals from ECOSAR-prioritized chlorinated aromatics and coupling products. Within the tested ratios, H_2_O_2_ neither accelerated SMX removal nor reduced the 10 min signals of the prioritized TPs. Among these products, TP31, TP32, and TP34 emerged as particularly relevant candidates for targeted monitoring because their ECOSAR prioritization coincided with high predicted water solubility and, for TP31 and TP32, substantial transport potential. TP1 and TP30 represented a different concern because of their greater predicted persistence and sorption. These findings indicate that SMX removal alone does not fully describe treatment performance and should be considered together with transformation-product behavior, mixture ecotoxicity, and environmental fate.

Two limitations should be considered when interpreting these findings: the use of a defined model matrix and the assessment of post-treatment biological responses at a single treatment time. The controlled design allowed the treatment systems to be compared under consistent conditions and helped isolate the effects of reagent composition. However, it does not reproduce the complexity of natural waters or show how biological responses may change during longer treatment. Future studies should therefore examine representative real-water matrices and extended treatment times to determine how these factors influence SMX degradation, transformation-product profiles, and post-treatment biological responses.

## Figures and Tables

**Table 1:** Pseudo-first-order kinetic parameters for SMX degradation in the investigated UV/NaOCl and UV/H_2_O_2_/NaOCl systems, with representative literature data for other treatment systems.

System	Pseudo-First-Order Rate Constant, k [min^−1^]	Coefficient of Determination (R^2^)	Details	Reference
SMX/NaOCl/UV (1:1)	0.215	0.998	All present-study systems: [SMX]_0_ = 0.4 mM; pH = 5; *T* = 20 °C; 150 W medium-pressure mercury lamp	This study
SMX/NaOCl/UV (1:2)	0.290	1.000		This study
SMX/NaOCl/UV (1:5)	0.342	0.999		This study
SMX/NaOCl/UV (1:10)	0.818	0.996		This study
SMX/H_2_O_2_/NaOCl/UV (1:5:10)	0.140	0.998		This study
SMX/H_2_O_2_/NaOCl/UV (1:10:5)	0.133	0.997		This study
SMX/UV	0.106	0.997	[SMX]_0_ = 0.4 mM; pH = 5; *T* = 20 °C; 150 W medium-pressure mercury lamp	[10]
SMX/H_2_O_2_/UV (1:1)	0.136	0.984		[10]
SMX/H_2_O_2_/UV (1:3)	0.130	0.993		[10]
SMX/H_2_O_2_/UV (1:15)	0.120	0.990		[10]
SMX/H_2_O_2_/UV (1:30)	0.132	0.987		[10]
SMX/Fe(II)/H_2_O_2_/UV (1:1:1)	0.186	0.981		[10]
SMX/Fe(II)/H_2_O_2_/UV (1:1:3)	0.262	0.994		[10]
SMX/Fe(II)/H_2_O_2_/UV (1:1.5:4)	0.316	0.995		[10]
SMX/Fe(II)/H_2_O_2_/UV (1:2:6)	0.709	0.975		[10]
SMX/NaOCl (1:10, fresh water)	0.08	0.925	[SMX]_0_ = 0.1 mM; *T* = 25 °C	[30]
SMX/NaOCl (1:10, marine culture water)	1.69	0.979		[30]
SMX/NaOCl (1:10, marine water)	3.44	0.984		[30]
SMX/H_2_O_2_/VUV (1:37)	0.510	0.9700	[SMX]_0_ = 0.04 mM; pH = 5.5; *T* = 25 °C; 10 W low-pressure vapor lamp with spectral output 90% at 254 nm and 10% at 185 nm	[35]
SMX/O_3_	0.45	0.98	[SMX]_0_ = 0.004 mM; pH 5.5; ozone-dose-normalized rate constant = 0.38 (mg⋅O_3_)^−1^	[39]
SMX/Fe(II)/H_2_O_2_/UV (1:0.5:10)	1.3079	-	[SMX]_0_ = 0.20 mM; pH = 3; *T* = 25 °C; 150 W medium-pressure mercury lamp	[43]
SMX/Na_2_S_2_O_8_/UV (1:63)	0.0283	0.9838	[SMX]_0_ = 0.08 mM; pH = 4; *T* = 25 °C; 8 W UV lamp	[44]
SMX/NaBrO_3_/UV (1:63)	0.0261	0.9727		[44]
SMX/H_2_O_2_/UV (1:63)	0.0271	0.9805		[44]
SMX/TiO_2_/UV	0.002	-	[SMX]_0_ = 0.004 mM; natural sunlight; photocatalyst dosage = 53.2 mg·L^−1^	[45]
SMX/Pt/TiO_2_/UV	0.02	-	[SMX]_0_ = 0.004 mM; natural sunlight; photocatalyst dosage = 28.7 mg·L^−1^	[45]
SMX/Pd/TiO_2_/UV	0.521	-	[SMX]_0_ = 0.004 mM; natural sunlight; photocatalyst dosage = 56.7 mg·L^−1^	[45]
SMX/β-Bi_2_O_3_ (flower-like)/UV	0.022	-	[SMX]_0_ = 0.040 mM; photocatalyst dosage = 2.0 g·L^−1^; initial pH = 3.0; room temperature; solar simulator 300 W	[46]
SMX/Oxone/Co_9_S_8_@S-N-RG	0.377	-	[SMX]_0_ = 0.040 mM; [Oxone]_0_ = 0.8 mM; cobalt sulfide–graphene doped with sulfur and nitrogen catalyst (Co_9_S_8_@S-N-RG) = 0.2 g⋅L^−1^	[47]
SMX/thermo-activated persulfate	<0.02	-	[SMX]_0_ = 0.03 mM; *T* = 30 °C	[48]
SMX/O_3_/ultrasounds	0.50	0.987	[SMX]_0_ = 0.4 mM; O_3_ dose = 3 g⋅h^−1^; pH = 9; *T* = 25 °C; ultrasound power density = 600 W⋅L^−1^	[49]

**Table 2:** The photodegradation conditions used in the study.

Reagents	SMX [mM]	H_2_O_2_ [mM]	NaOCl [mM]
SMX/NaOCl/UV (1:1)	0.4	-	0.4
SMX/NaOCl/UV (1:2)	0.4	-	0.8
SMX/NaOCl/UV (1:5)	0.4	-	2
SMX/NaOCl/UV (1:10)	0.4	-	4
SMX/H_2_O_2_/NaOCl/UV (1:5:10)	0.4	2	4
SMX/H_2_O_2_/NaOCl/UV (1:10:5)	0.4	4	2

## Data Availability

The data presented in this study are available in the article and Supplementary Materials. Additional data are available from the corresponding author upon reasonable request.

## Supplementary Materials

The following supporting information can be downloaded at https://www.mdpi.com/article/10.3390/molecules31183220/s1. Supplementary_Data.xlsx: LC-MS-assigned transformation products, predicted biological and toxicological endpoints, physicochemical descriptors, and environmental fate outputs; Supplementary_Chromatograms: Representative LC–MS chromatograms supporting chromatographic monitoring of SMX and the assigned transformation products TP1–TP34.
