# Supplementary material for: UV/Chlorine Treatment of Sulfamethoxazole: Removal, Transformation Products, Ecotoxicity, and Environmental Fate
Source: Molecules. 2026 Sep 12;31(18):3220. doi: 10.3390/molecules31183220 (PMC13608921; doi:10.3390/molecules31183220)
Supplement: Supplementary file 1 [file molecules-31-03220-s001.zip › Supplementary_Chromatograms.pdf]

SUPPLEMENTARY MATERIALS

UV/Chlorine Treatment of Sulfamethoxazole: Removal, Transformation Products, Ecotoxicity, and Environmental Fate

Waldemar Studziński[1]*, Alicja Gackowska[1], Edyta Kudlek[2], Maciej Przybyłek[3,4]

[1]*Department of Food Analysis and Environmental Protection, Faculty of Chemical Technology and Engineering, Bydgoszcz University of Science and Technology, Seminaryjna 3, 85-326 Bydgoszcz, Poland; alicja.gackowska@pbs.edu.pl (A.G.)*
[2]*Department of Water and Wastewater Engineering, Faculty of Energy and Environmental Engineering, Silesian University of Technology, Konarskiego 18, 44-100 Gliwice, Poland; edyta.kudlek@polsl.pl*
[3]*Department of Physical Chemistry, Faculty of Pharmacy, Nicolaus Copernicus University in Toruń, Ludwik Rydygier Collegium Medicum in Bydgoszcz, Kurpińskiego 5, 85-950 Bydgoszcz, Poland; m.przybylek@cm.umk.pl*
[4]*Institute of Advanced Studies, Nicolaus Copernicus University in Toruń, Wileńska 4, 87-100 Toruń, Poland*
** Correspondence: Waldemar.Studzinski@pbs.edu.pl*

Table S1. Representative chromatograms and key identification data for sulfamethoxazole (SMX) and transformation products TP1–TP34. The left column lists the compound name, SMILES, m/z, and retention time; shared chromatograms are repeated in the relevant rows where needed.

Transformation product	Chromatogram
**SMX**<br>**Name:** 4-amino-N-(5-methyl-1,2-oxazol-3-yl)benzene-1-sulfonamide<br>**SMILES:** CC1=CC(=NO1)NS(=O)(=O)C2=CC=C(C=C2)N<br>**m/z:** 254 [M+H]$^+$<br>t_R: 3.813 min	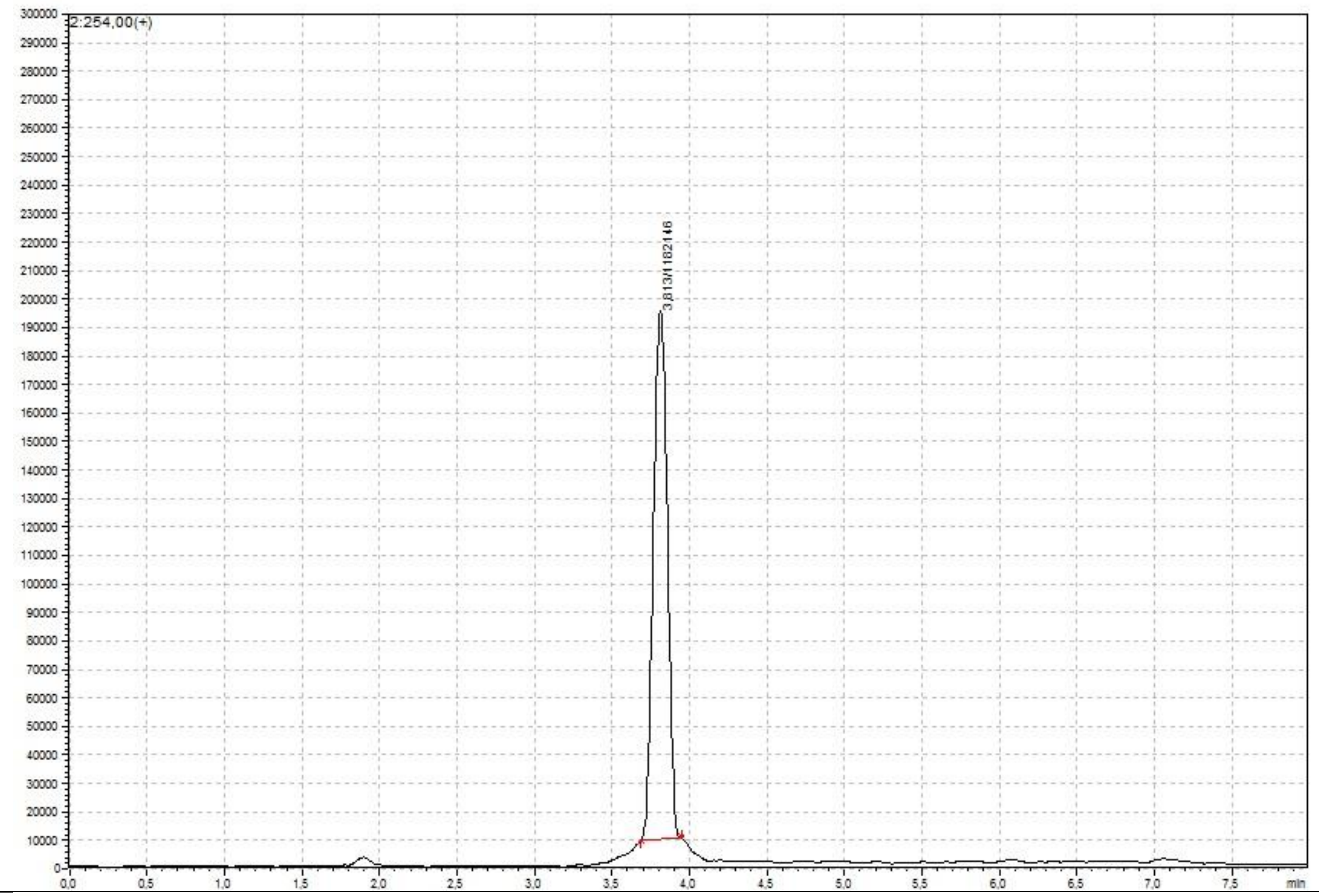
**TP1**<br>**Name:** N-(5-methyl-1,2-oxazol-3-yl)-4-(2-{4-[(5-methyl-1,2-oxazol-3-yl)sulfamoyl]phenyl}diazen-1-yl)benzene-1-sulfonamide<br>**SMILES:** CC1=CC(NS(=O)(=O)C2=CC=C(C=C2)N=NC2=CC=C(C=C2)S(=O)(=O)NC2=NOC(C)=C2)=NO1<br>**m/z:** 503 [M+H]$^+$<br>t_R: 4.136 min	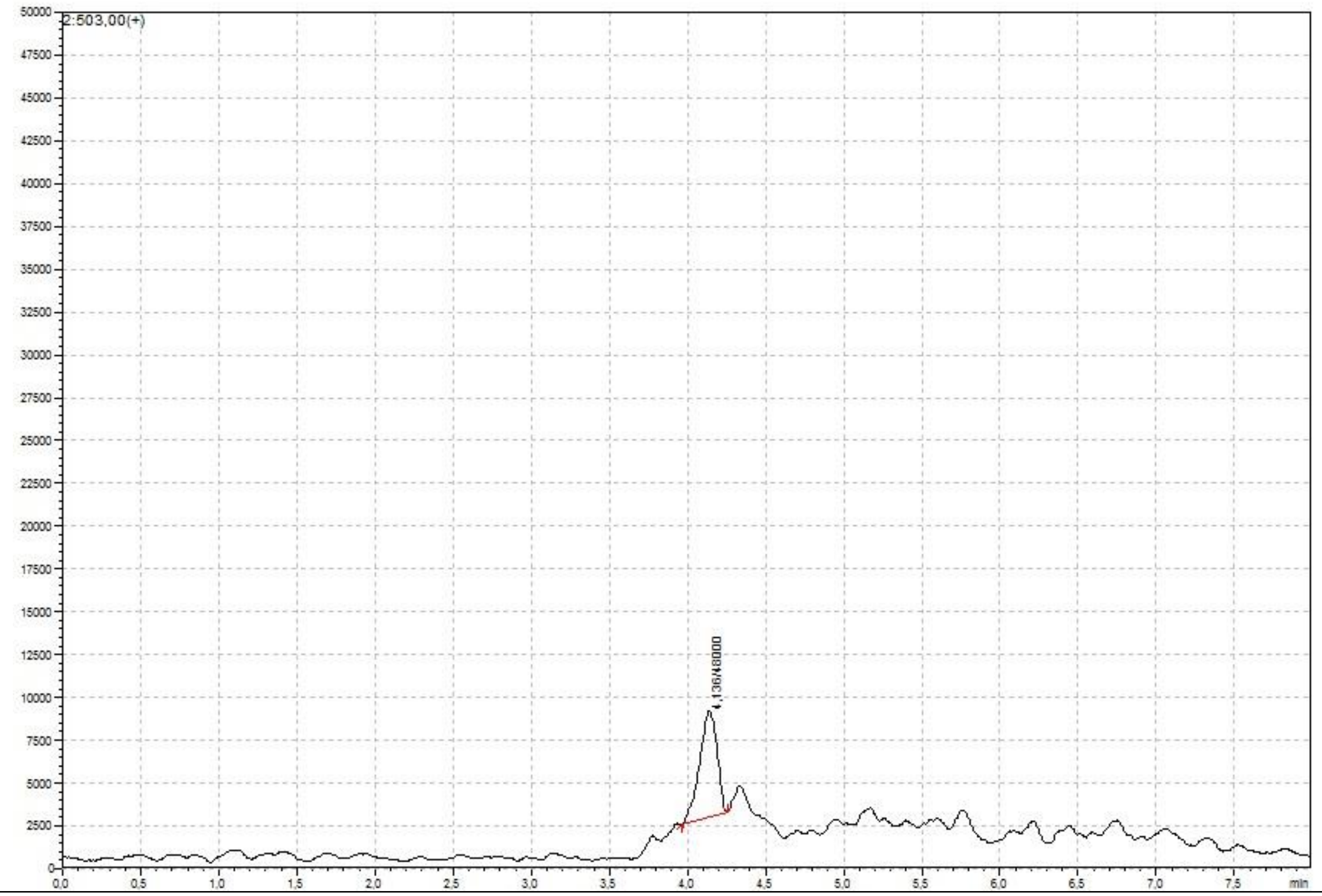

Transformation product	Chromatogram
**TP2**<br>**Name:** 4-amino-N-(5-formyl-1,2-oxazol-3-yl)benzene-1-sulfonamide<br>**SMILES:** NC1=CC=C(C=C1)S(=O)(=O)NC1=NOC(C=O)=C1<br>**m/z:** 267 $[M]^+$<br>t_R**:** 3.842 min	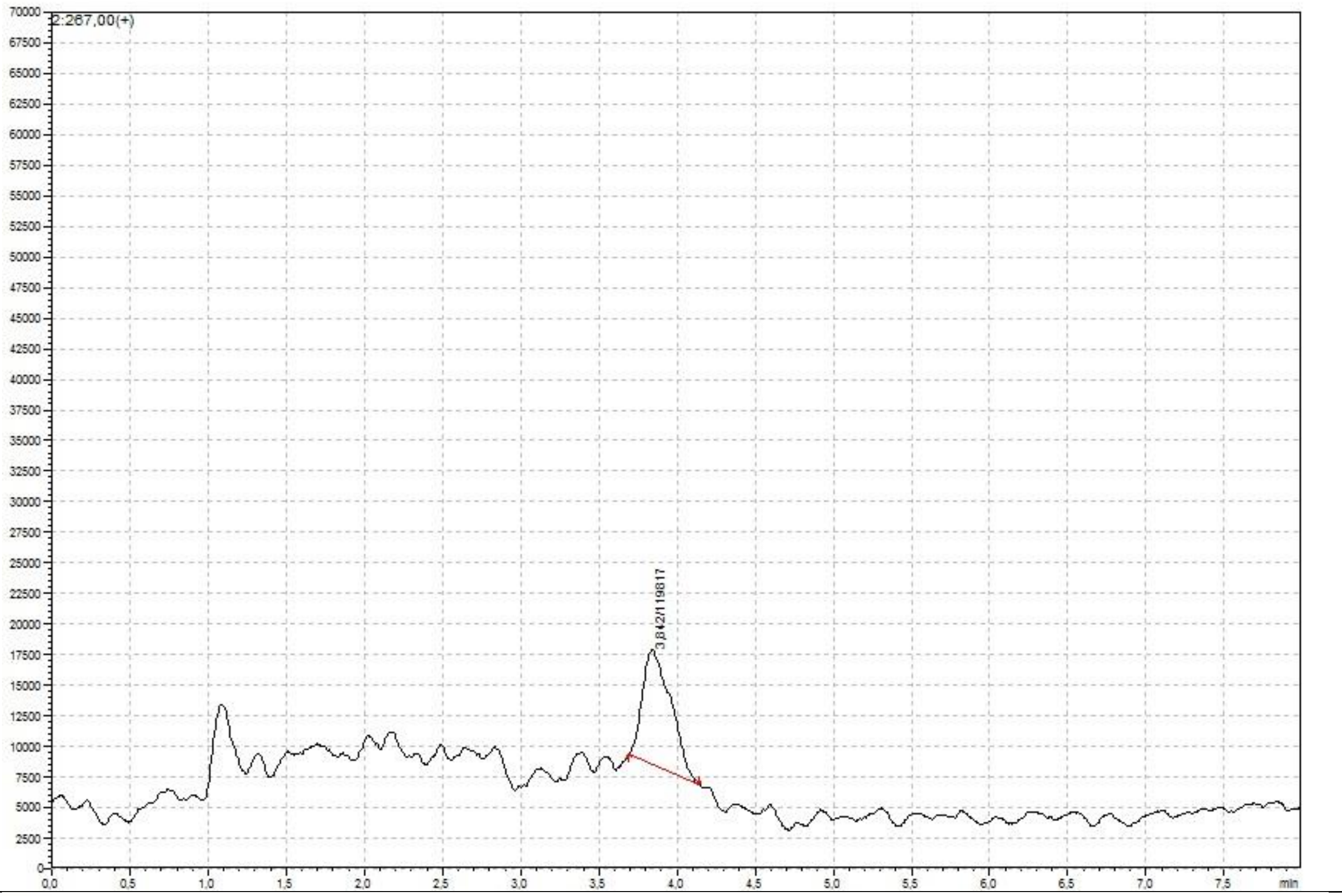
**TP3**<br>**Name:** N-(5-methyl-1,2-oxazol-3-yl)sulfamic acid<br>**SMILES:** CC1=CC(NS(O)(=O)=O)=NO1<br>**m/z:** 178 $[M]^+$<br>t_R**:** 4.129 min	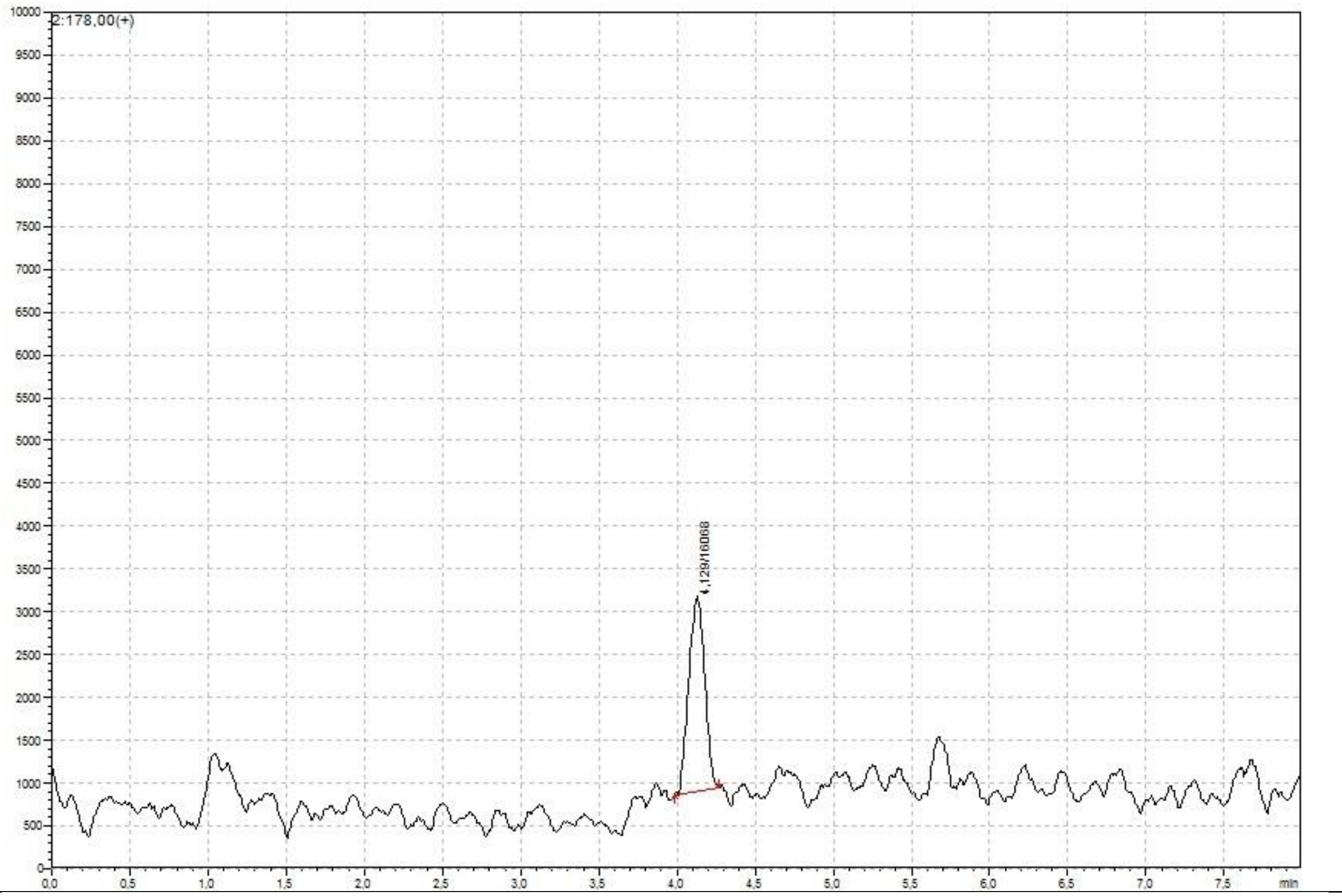
**TP4**<br>**Name:** 4-amino-3-chloro-N-(5-methyl-1,2-oxazol-3-yl)benzene-1-sulfonamide<br>**SMILES:** CC1=CC(NS(=O)(=O)C2=CC(Cl)=C(N)C=C2)=NO1<br>**m/z:** 288 $[M+H]^+$ (^{35}Cl); 290 $[M+H]^+$ (^{37}Cl)<br>t_R**:** 4.538 min	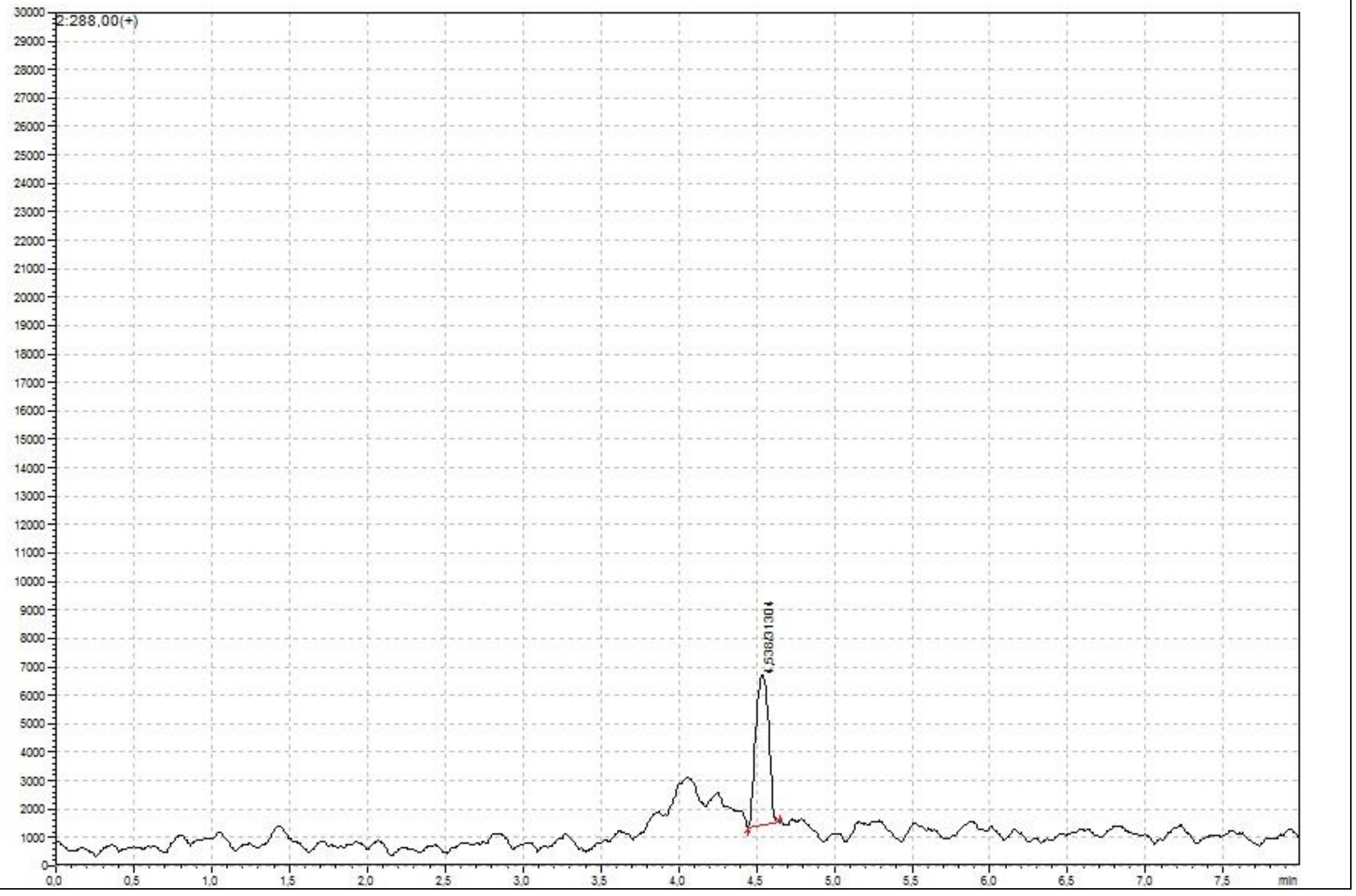

Transformation product	Chromatogram
**TP5**<br>**Name:** 4-amino-3-hydroxy-N-(5-methyl-1,2-oxazol-3-yl)benzene-1-sulfonamide<br>**SMILES:**<br>CC1=CC(NS(=O)(=O)C2=CC(O)=C(N)C=C2)=NO1<br>**m/z:** 269 $[M]^+$<br>**t_R:** 3.886 min	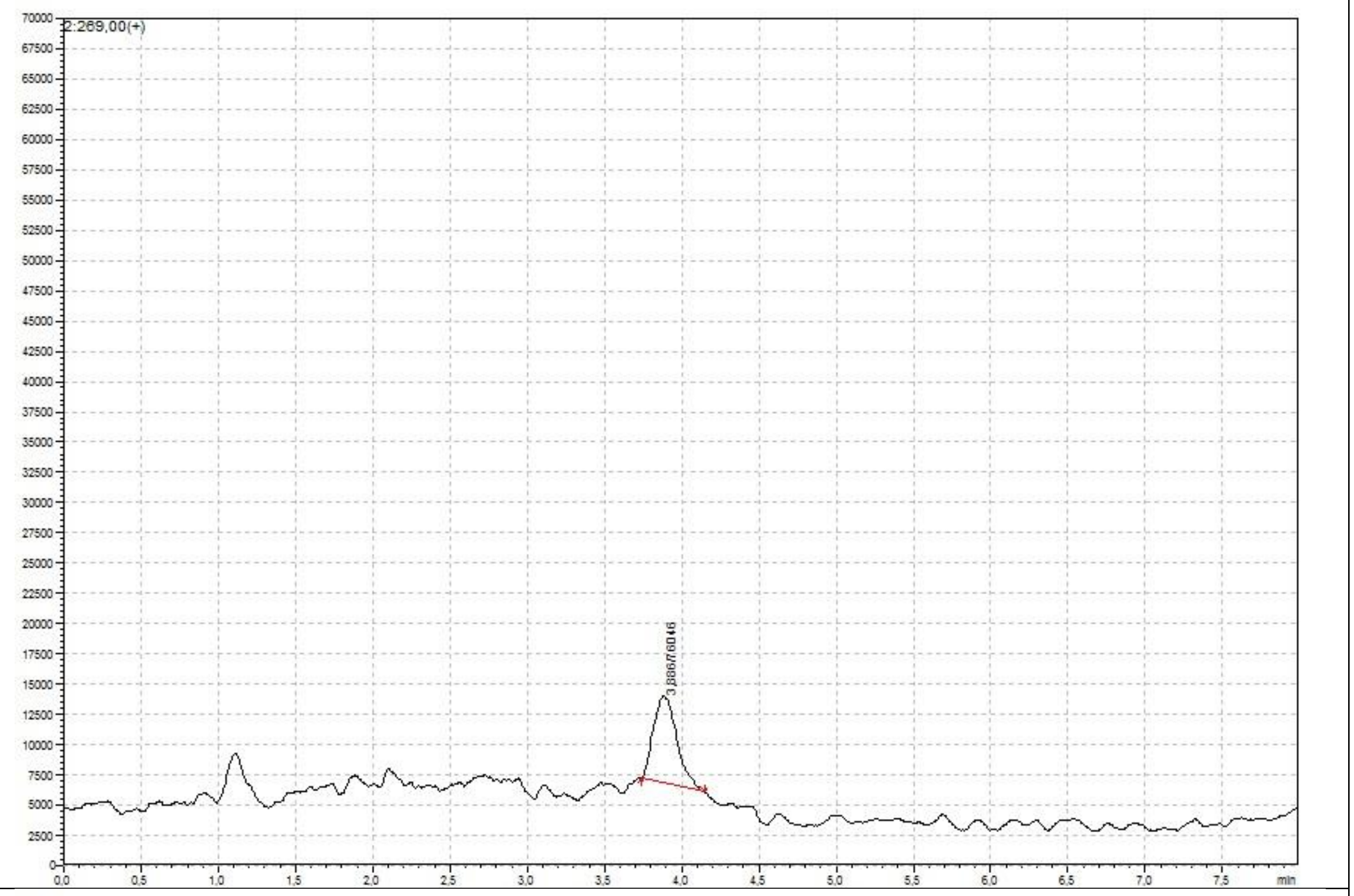
**TP6**<br>**Name:** 4-aminobenzene-1-sulfonic acid<br>**SMILES:** C1=CC(=CC=C1N)S(=O)(=O)O<br>**m/z:** 173 $[M]^+$<br>**t_R:** 4.126 min	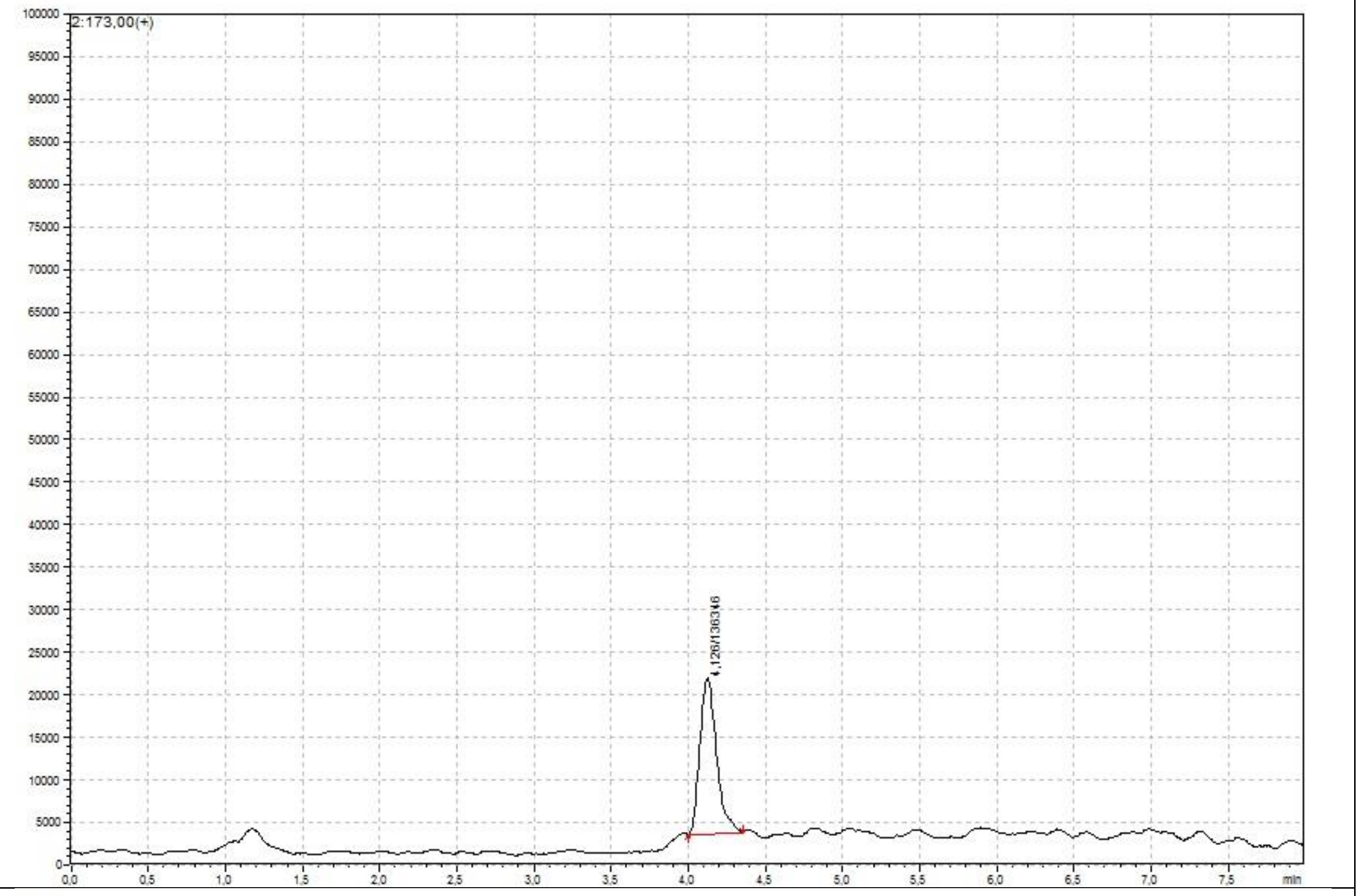
**TP7**<br>**Name:** (1S,2S,3S)-1-amino-S-(4-aminophenyl)-2,3-dihydroxybutane-1-sulfonamido<br>**SMILES:**<br>C[C@H](O)[C@@H](O)[C@@H](N)NS(=O)(=O)C1=CC=C(N)C=C1<br>**m/z:** 276 $[M+H]^+$<br>**t_R:** 3.811 min	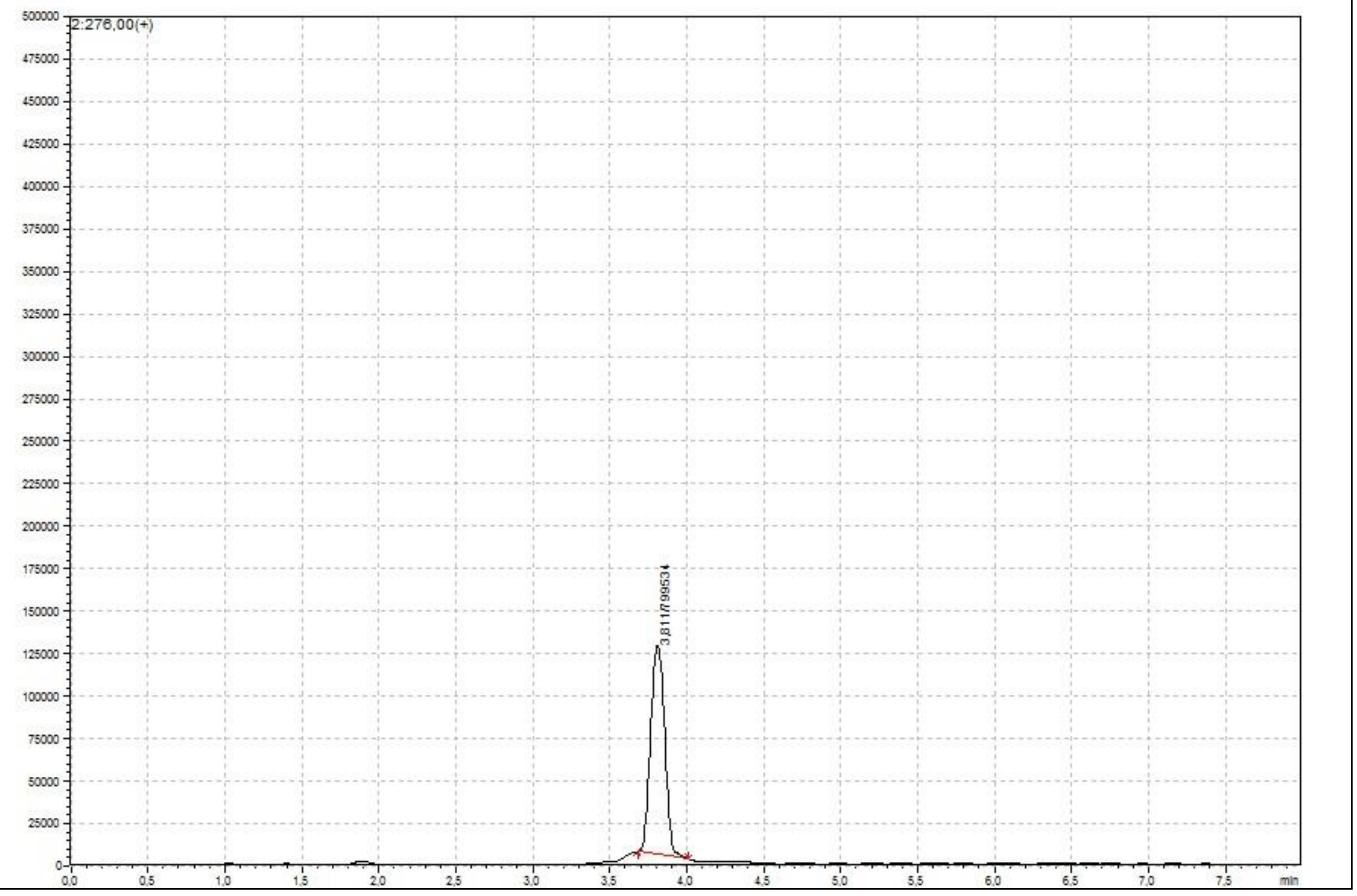

Transformation product	Chromatogram
**TP8**<br>**Name:** N-(5-methyl-1,2-oxazol-3-yl)-4-nitrosobenzene-1-sulfonamide<br>**SMILES:**<br>CC1=CC(NS(=O)(=O)C2=CC=C(C=C2)N=O)=NO1<br>**m/z:** 267 $[M]^+$<br>t_R: 3.842 min	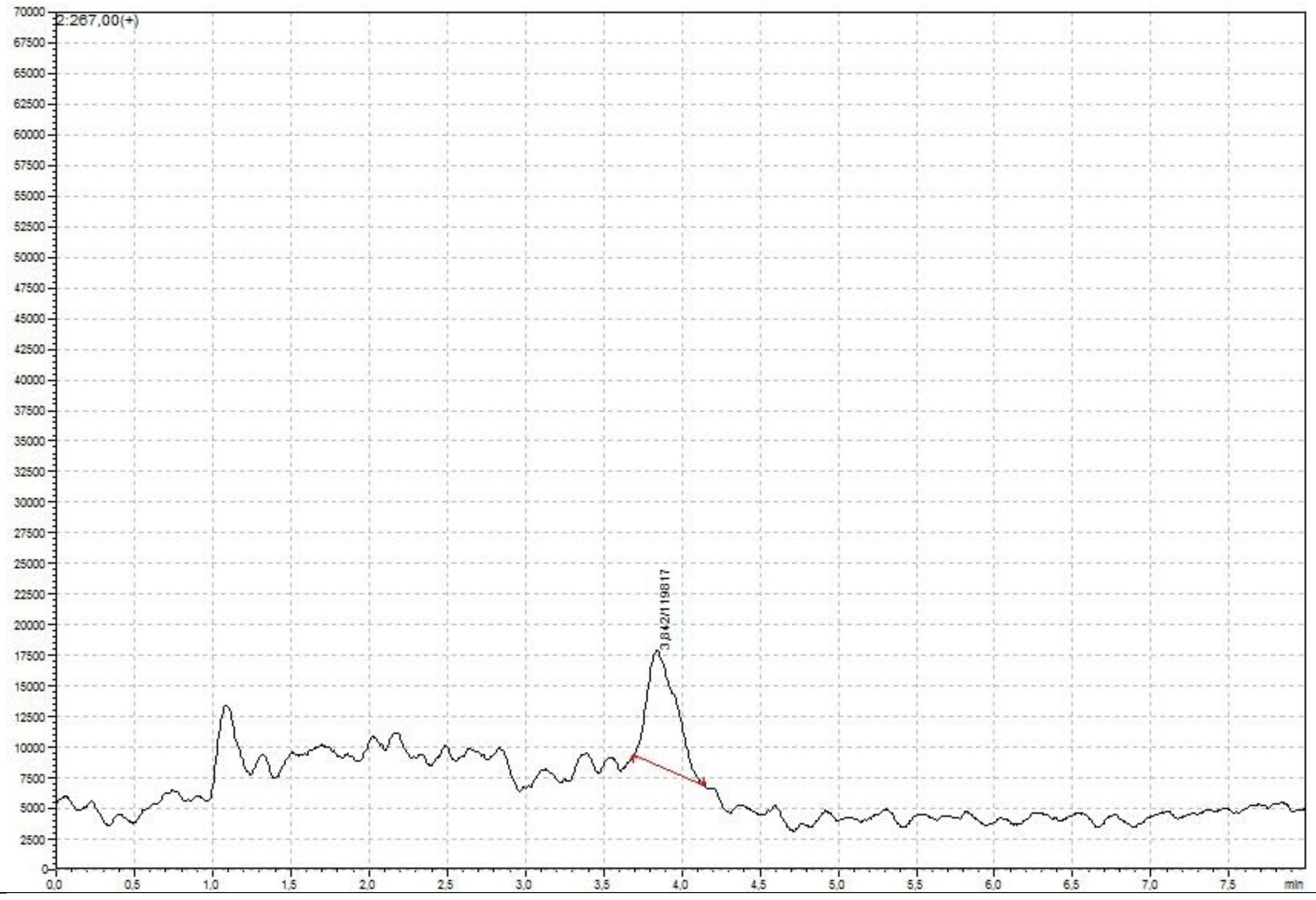
**TP9**<br>**Name:** 4-amino-3-chloro-N-(5-formyl-1,2-oxazol-3-yl)benzene-1-sulfonamide<br>**SMILES:**<br>NC1=C(Cl)C=C(C=C1)S(=O)(=O)NC1=NOC(C=O)=C1<br>**m/z:** 301 $[M]^+$ (^{35}Cl); 303 $[M]^+$ (^{37}Cl)<br>t_R: 5.578 min	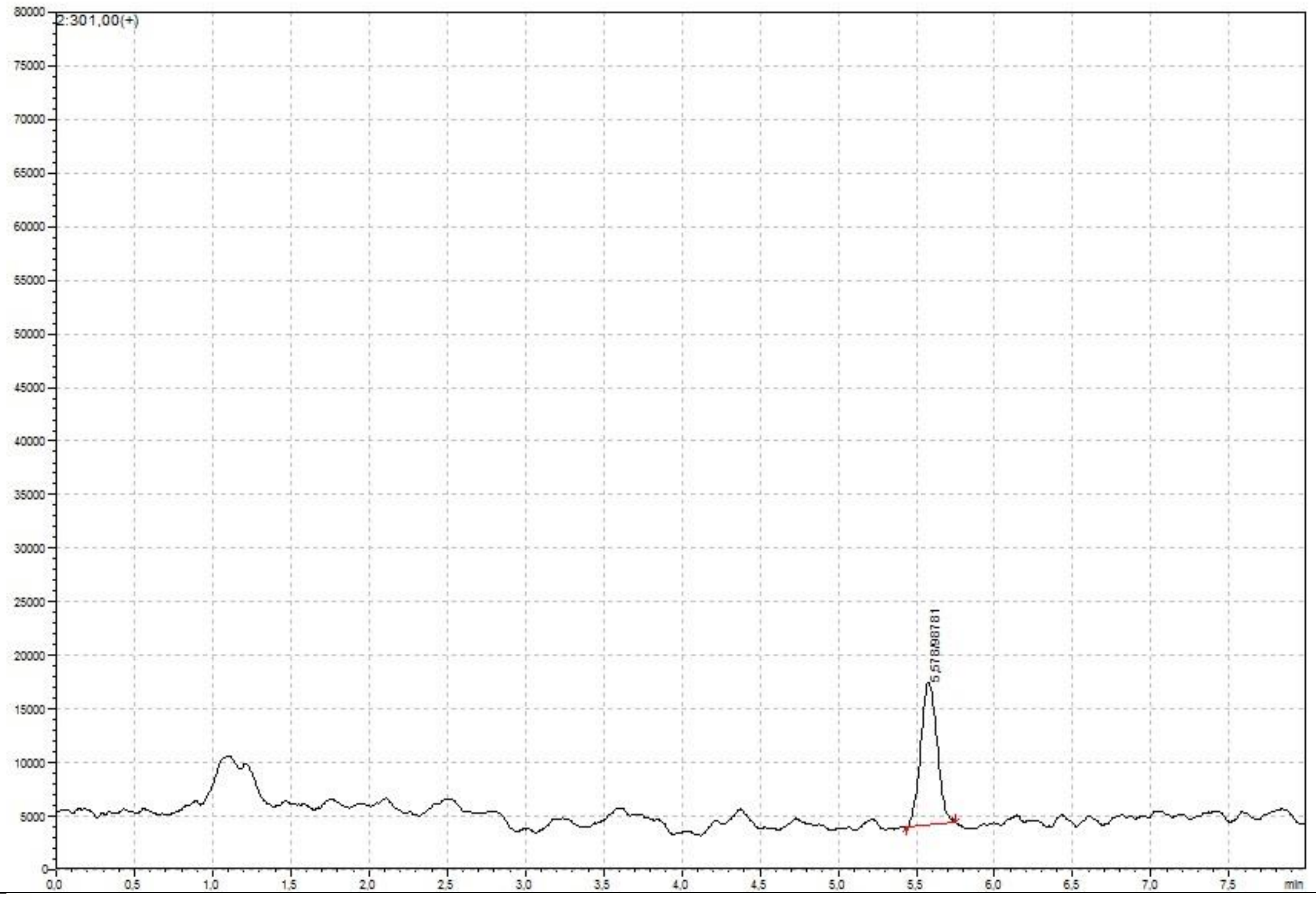
**TP10**<br>**Name:** N-(4-chloro-5-methyl-1,2-oxazol-3-yl)sulfamic acid<br>**SMILES:**<br>CC1=C(Cl)C(NS(O)(=O)=O)=NO1<br>**m/z:** 214 $[M+H]^+$ (^{35}Cl); 216 $[M+H]^+$ (^{37}Cl)<br>t_R: 4.134 min	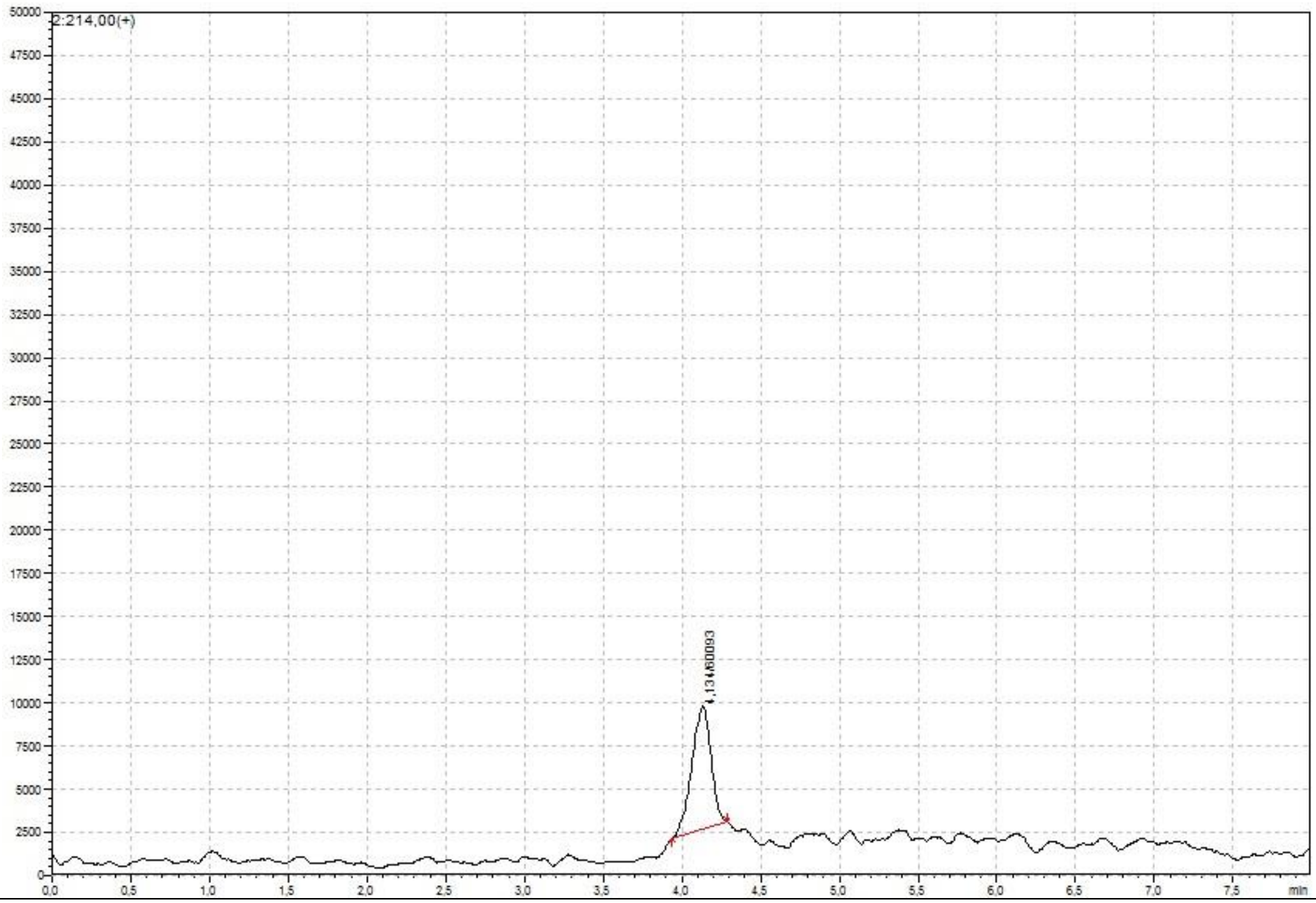

Transformation product	Chromatogram
**TP11**<br>**Name:** 5-methyl-1,2-oxazol-3-amine<br>**SMILES:** CC1=CC(=NO1)N<br>**m/z:** 99 $[M+H]^+$<br>t_R**:** 1.565 min	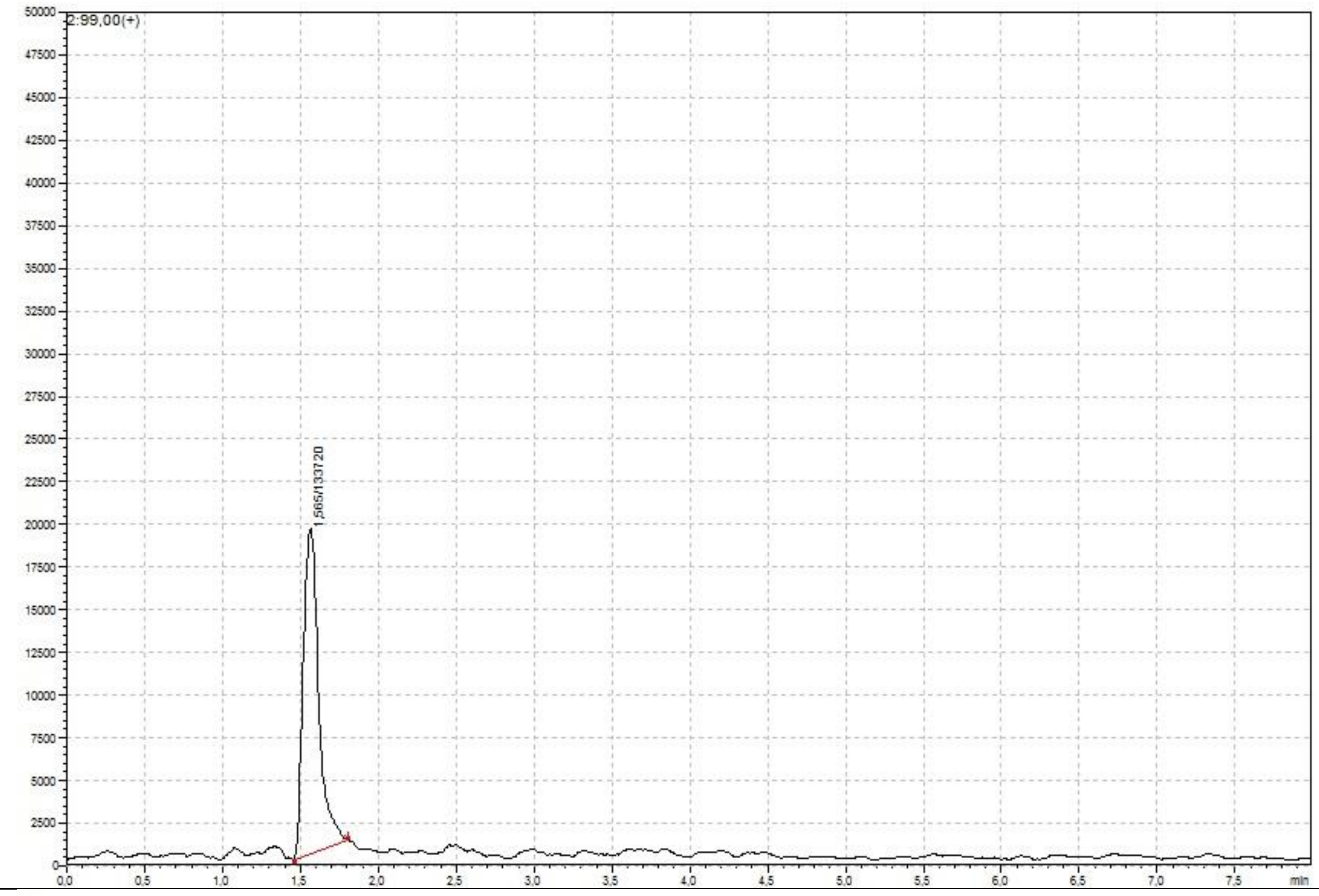
**TP12**<br>**Name:** 4-amino-3,5-dichloro-N-(5-methyl-1,2-oxazol-3-yl)benzene-1-sulfonamide<br>**SMILES:**<br>CC1=CC(NS(=O)(=O)C2=CC(Cl)=C(N)C(Cl)=C2)=NO1<br>**m/z:** 322 $[M+H]^+$ ($^{35}Cl_2$); 324 $[M+H]^+$ (^{35}Cl ^{37}Cl); 326 $[M+H]^+$ ($^{37}Cl_2$)<br>t_R**:** 4.082 min	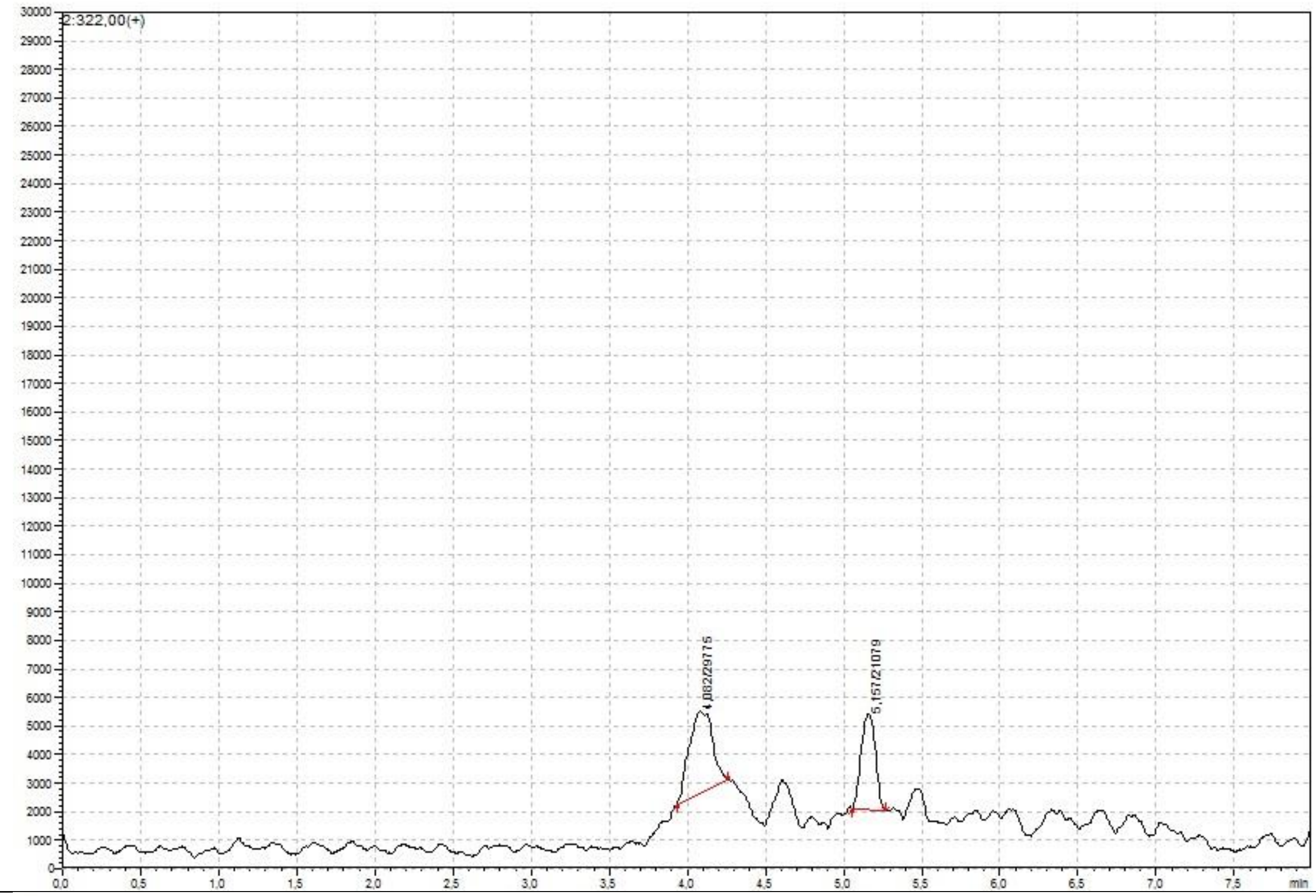
**TP13**<br>**Name:** 4-amino-3-chloro-N-[(4S,5R)-4,5-dihydroxy-5-methyl-4,5-dihydro-1,2-oxazol-3-yl]benzene-1-sulfonamide<br>**SMILES:**<br>C[C@@]1(O)ON=C(NS(=O)(=O)C2=CC(Cl)=C(N)C=C2)[C@@H]1O<br>**m/z:** 322 $[M+H]^+$ (^{35}Cl); 324 $[M+H]^+$ (^{37}Cl)<br>t_R**:** 5.157 min	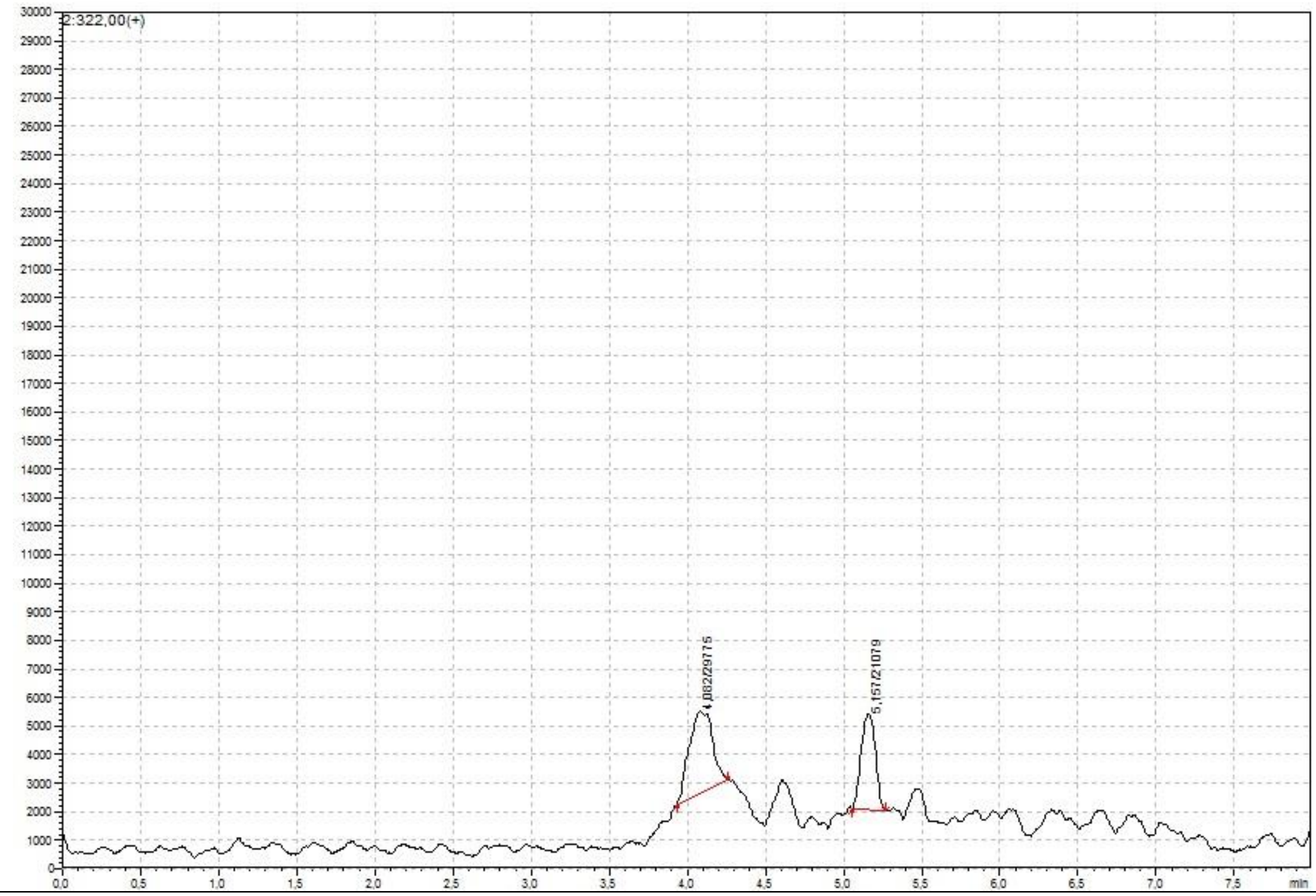

Transformation product	Chromatogram
**TP14**<br>**Name:** 4-amino-3-chloro-5-hydroxy-N-(5-methyl-1,2-oxazol-3-yl)benzene-1-sulfonamide<br>**SMILES:** CC1=CC(NS(=O)(=O)C2=CC(O)=C(N)C(Cl)=C2)=NO1<br>**m/z:** 304 $[M+H]^+$ (^{35}Cl); 306 $[M+H]^+$ (^{37}Cl)<br>$\mathbf{t_R}$**:** 3.913 min	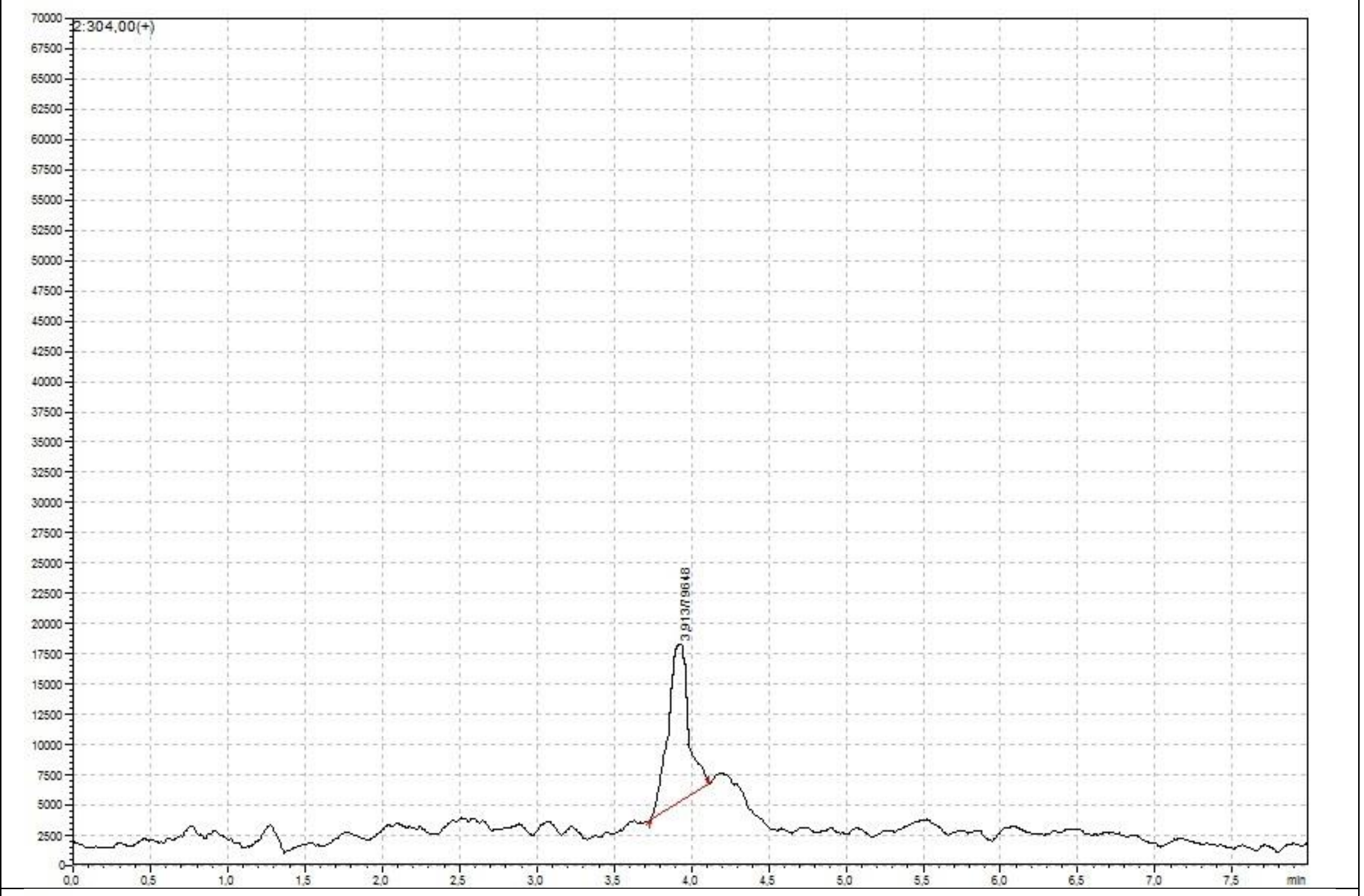
**TP15**<br>**Name:** benzenesulfinic acid<br>**SMILES:** OS(=O)C1=CC=CC=C1<br>**m/z:** 143 $[M+H]^+$<br>$\mathbf{t_R}$**:** 1.029 min	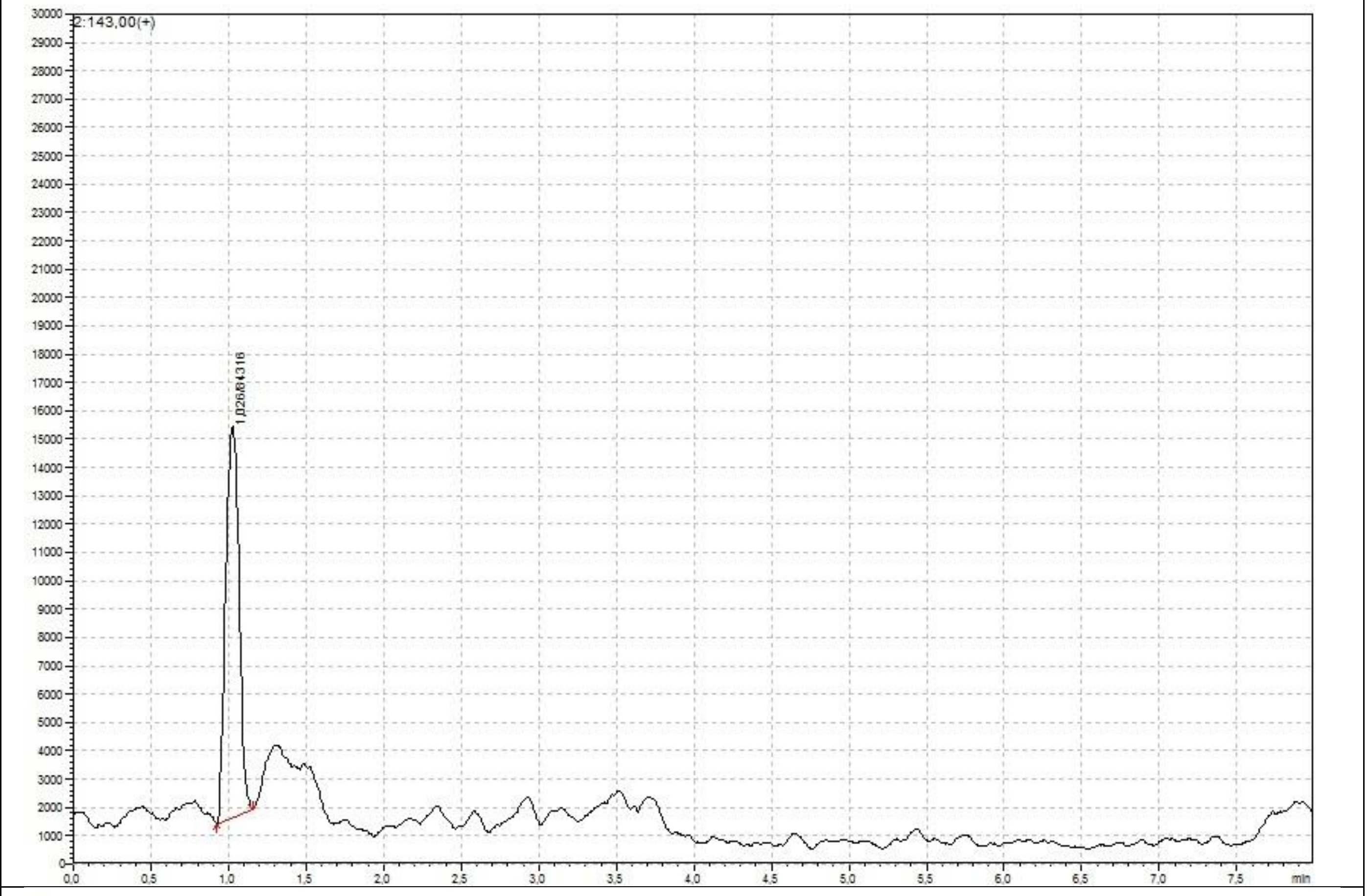
**TP16**<br>**Name:** 4-aminophenol<br>**SMILES:** NC1=CC=C(O)C=C1<br>**m/z:** 109 $[M]^+$<br>$\mathbf{t_R}$**:** 1.033 min	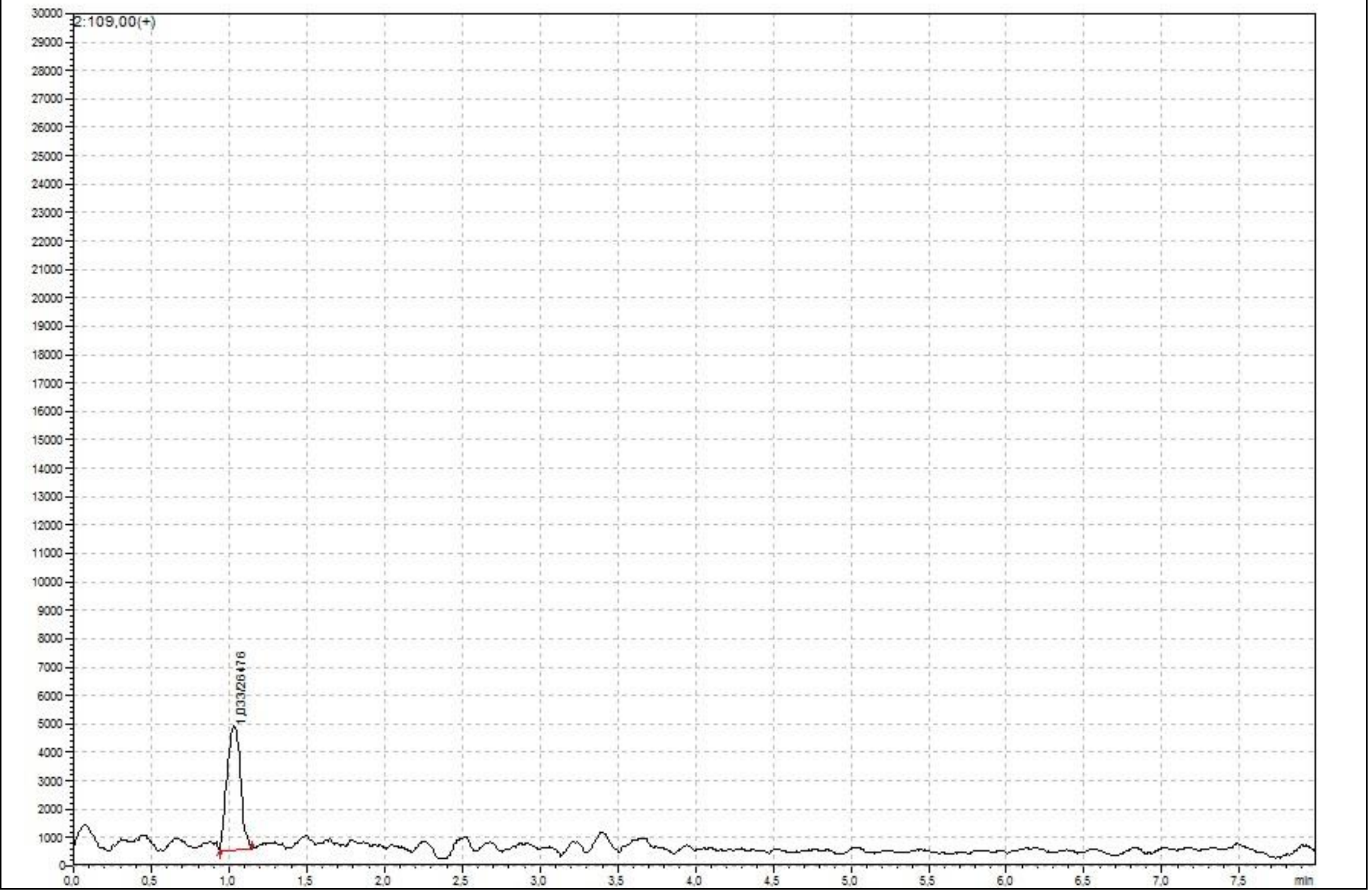

Transformation product	Chromatogram
**TP17**<br>**Name:** 4-sulfonylaniline<br>**SMILES:** NC1=CC=C(C=C1)S(=O)=O<br>**m/z:** 156 $[M]^+$<br>$\mathbf{t_R}$**:** 3.818 min	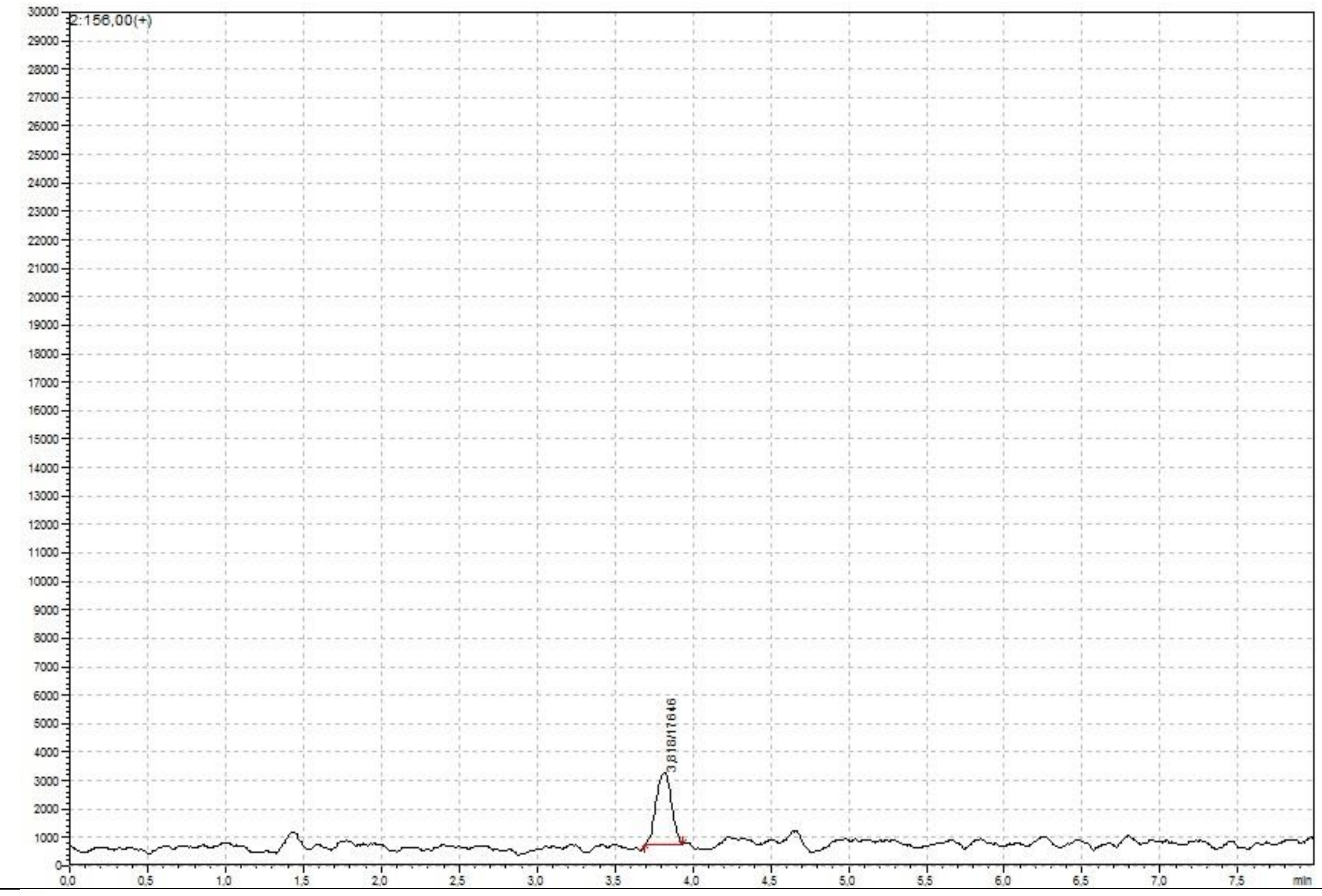
**TP18**<br>**Name:** (1S,2S,3S)-1-amino-S-(4-amino-3-hydroxyphenyl)-2,3-dihydroxybutane-1-sulfonamido<br>**SMILES:**<br>C[C@H](O)[C@@H](O)[C@@H](N)NS(=O)(=O)C1=CC=C(N)C(O)=C1<br>**m/z:** 292 $[M+H]^+$<br>$\mathbf{t_R}$**:** 3.811 min	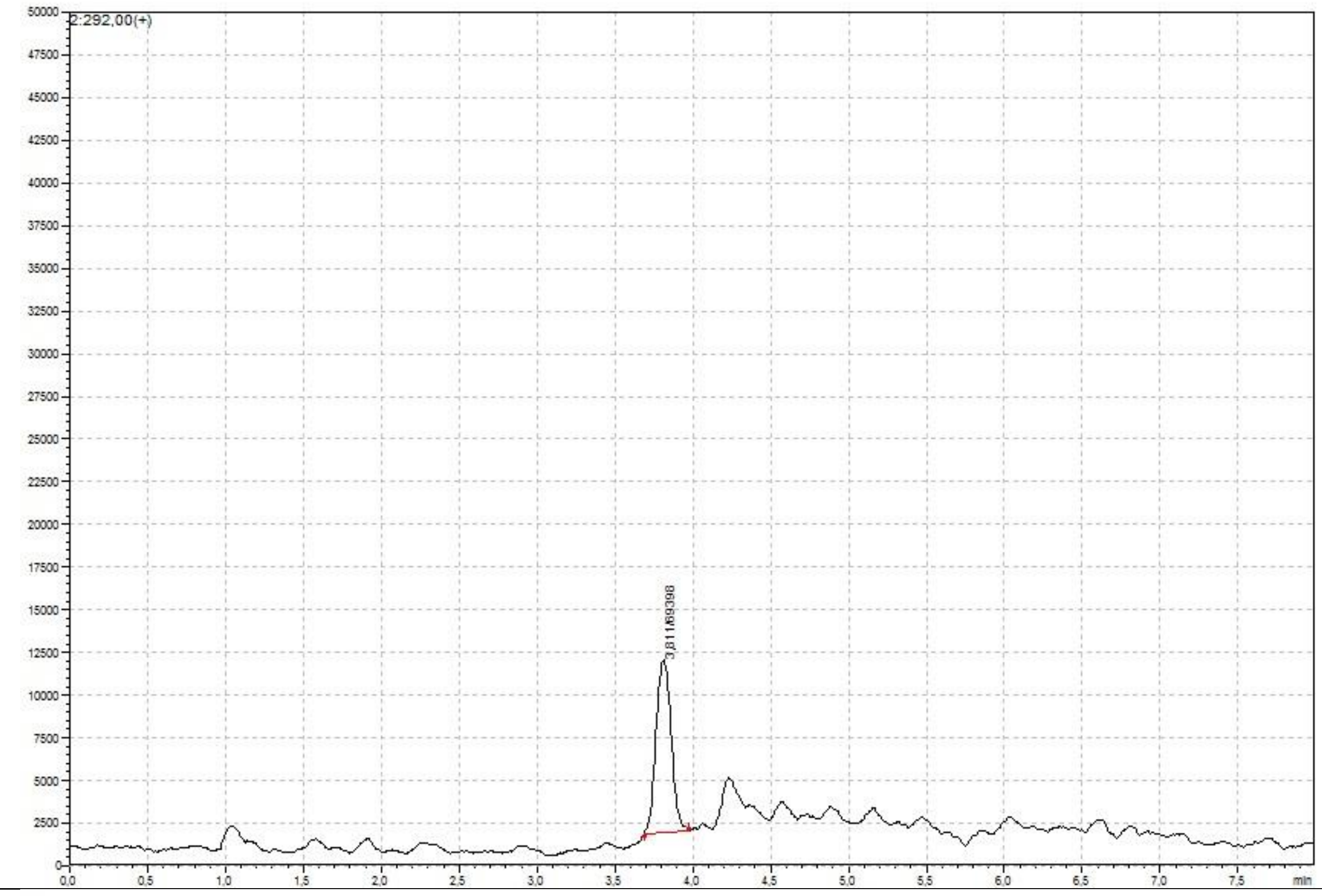
**TP19**<br>**Name:** 3-chloro-N-(5-methyl-1,2-oxazol-3-yl)-4-nitrosobenzene-1-sulfonamide<br>**SMILES:**<br>CC1=CC(NS(=O)(=O)C2=CC=C(N=O)C(Cl)=C2)=NO1<br>**m/z:** 301 $[M]^+$ (^{35}Cl); 303 $[M]^+$ (^{37}Cl)<br>$\mathbf{t_R}$**:** 5.578 min	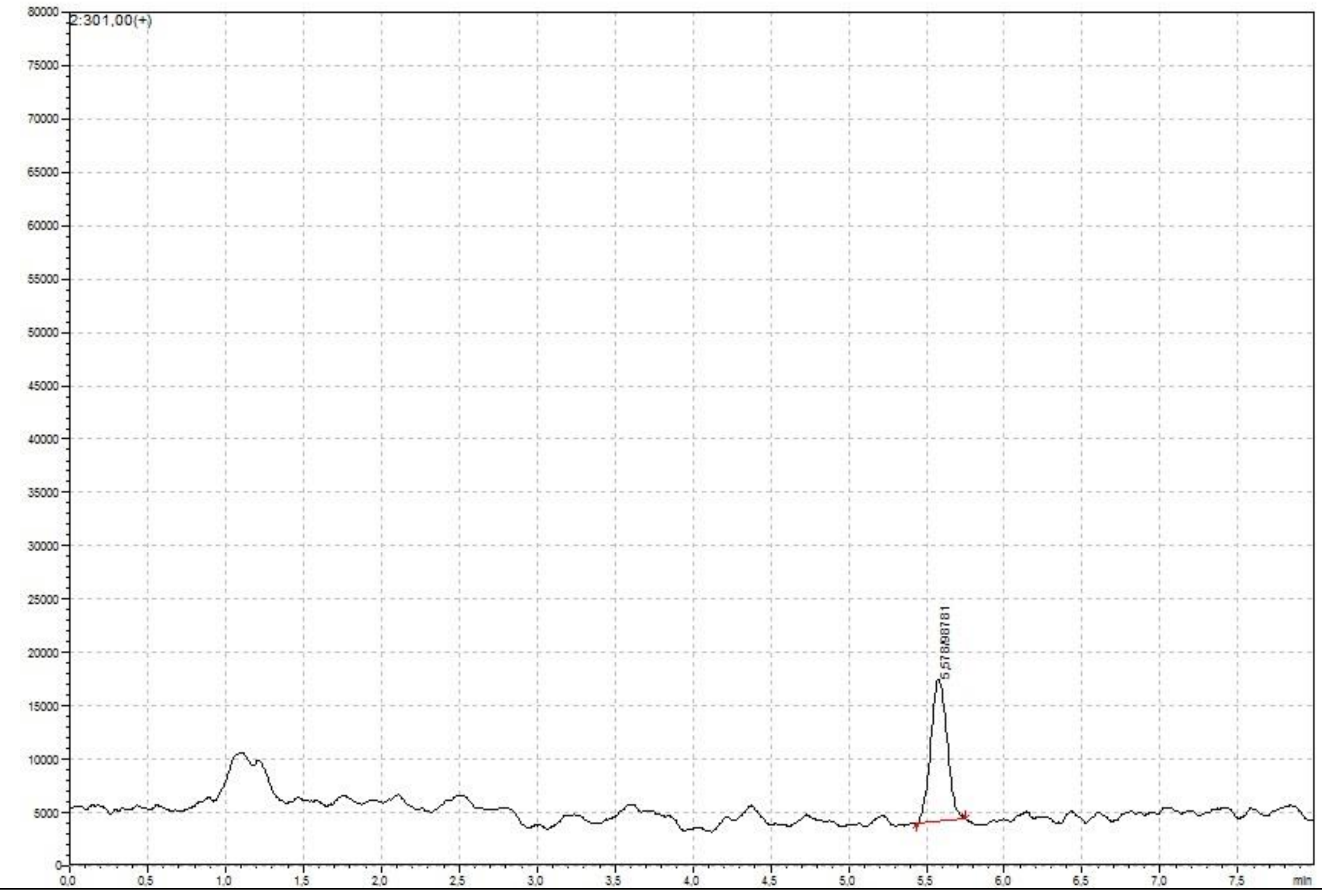

<table>
<tr><th>Transformation product</th><th>Chromatogram</th></tr>
<tr><td>TP20
Name: 3-(4-amino-3-chlorobenzenesulfonamido)-1,2-oxazole-5-carboxylic acid
SMILES:
NC1=C(Cl)C=C(C=C1)S(=O)(=O)NC1=NOC(=C1)C(O)=O
m/z: 318 $[M+H]^+$ (^{35}Cl); 320 $[M+H]^+$ (^{37}Cl)
$\mathbf{t_R}$: 3.813 min</td><td>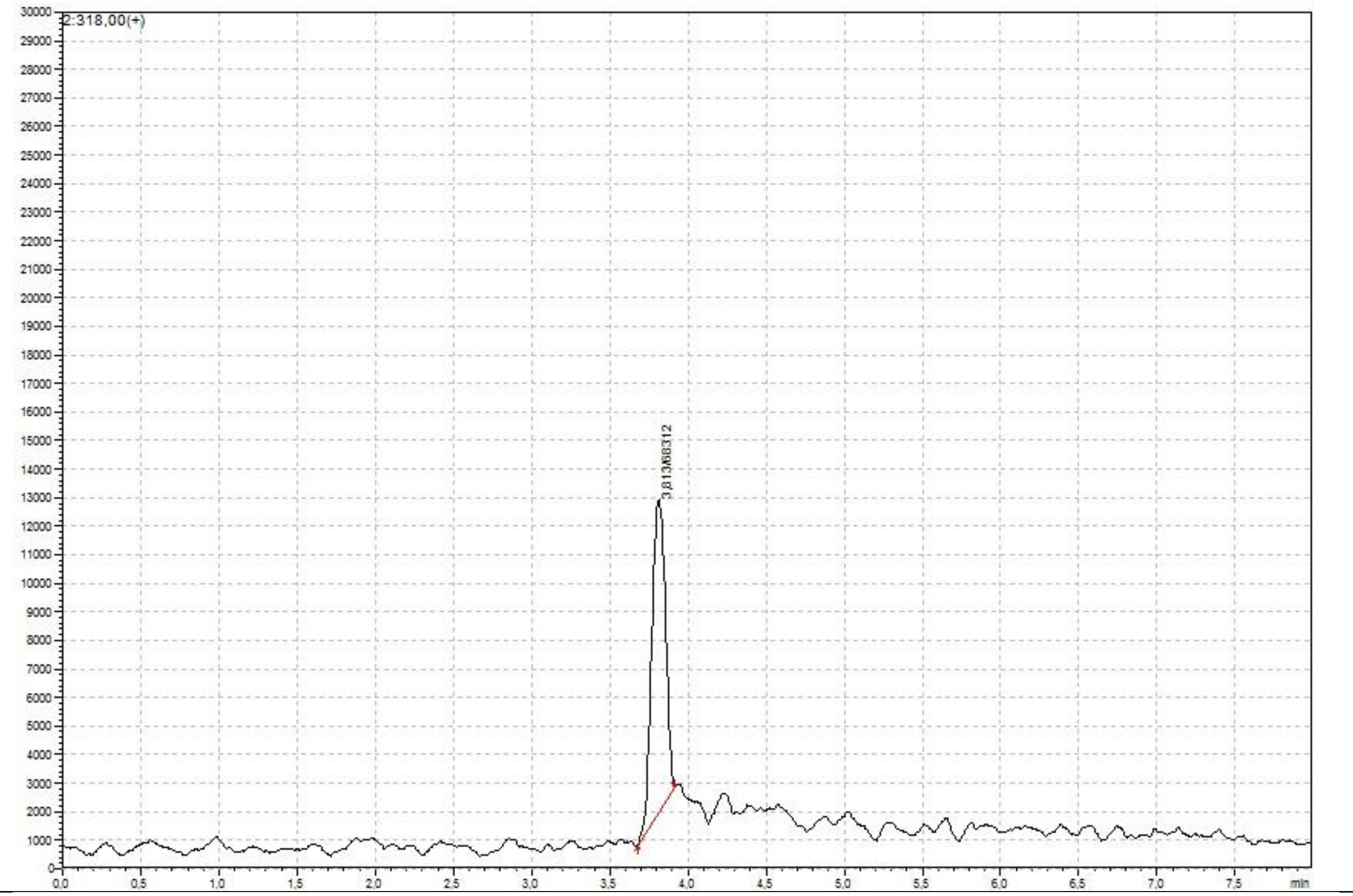
</td></tr>
<tr><td>TP21
Name: 5-methyl-3-[2-(5-methyl-1,2-oxazol-3-yl)diazen-1-yl]-1,2-oxazol-4-ol
SMILES:
CC1=CC(=NO1)N=NC2=NOC(=C2O)C
m/z: 209 $[M+H]^+$
$\mathbf{t_R}$: 4.441 min</td><td>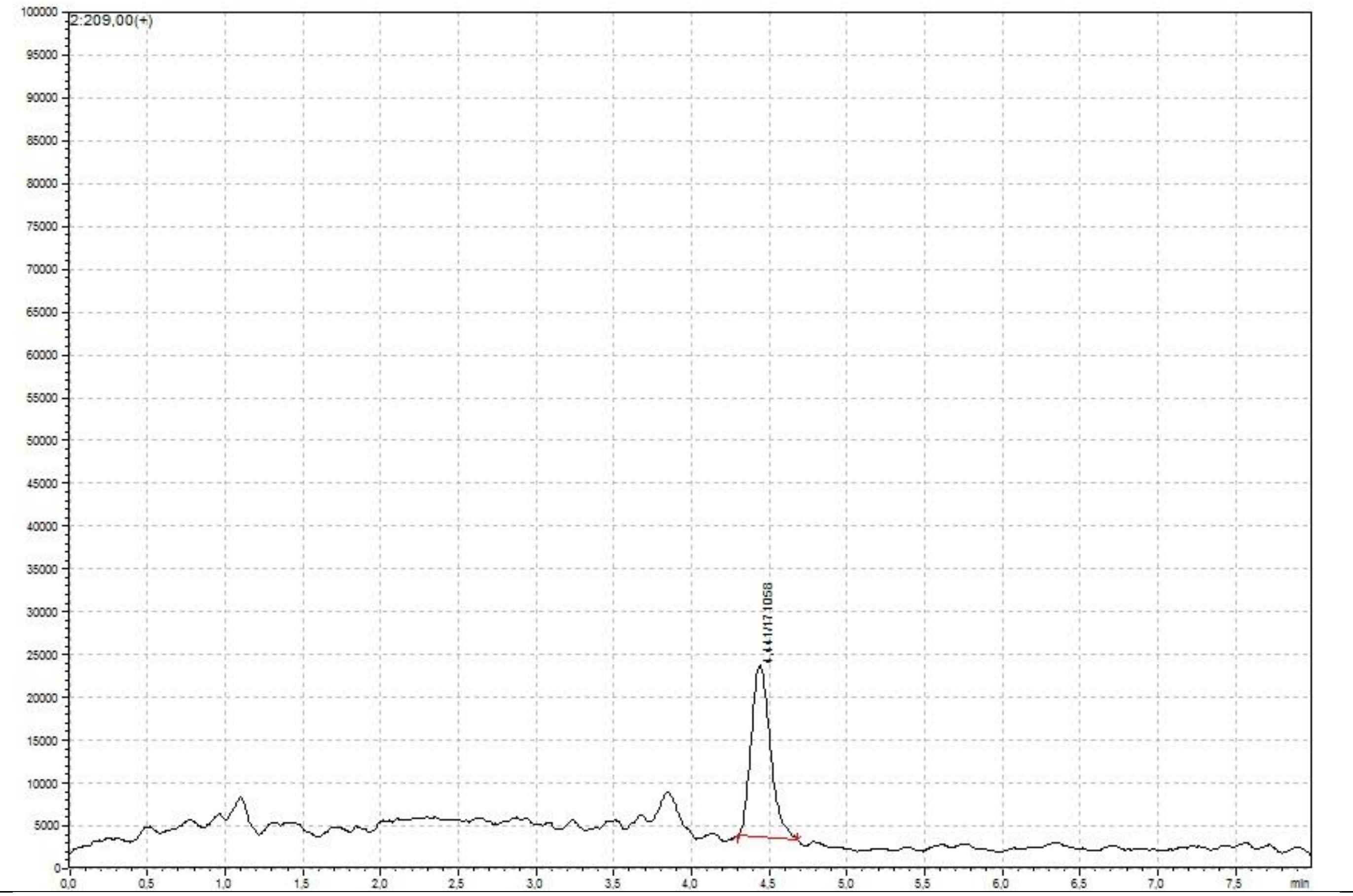
</td></tr>
<tr><td>TP22
Name: 4-chloro-5-methyl-1,2-oxazol-3-amine
SMILES: CC1=C(Cl)C(=NO1)N
m/z: 133 $[M+H]^+$ (^{35}Cl); 135 $[M+H]^+$ (^{37}Cl)
$\mathbf{t_R}$: 1.021 min</td><td>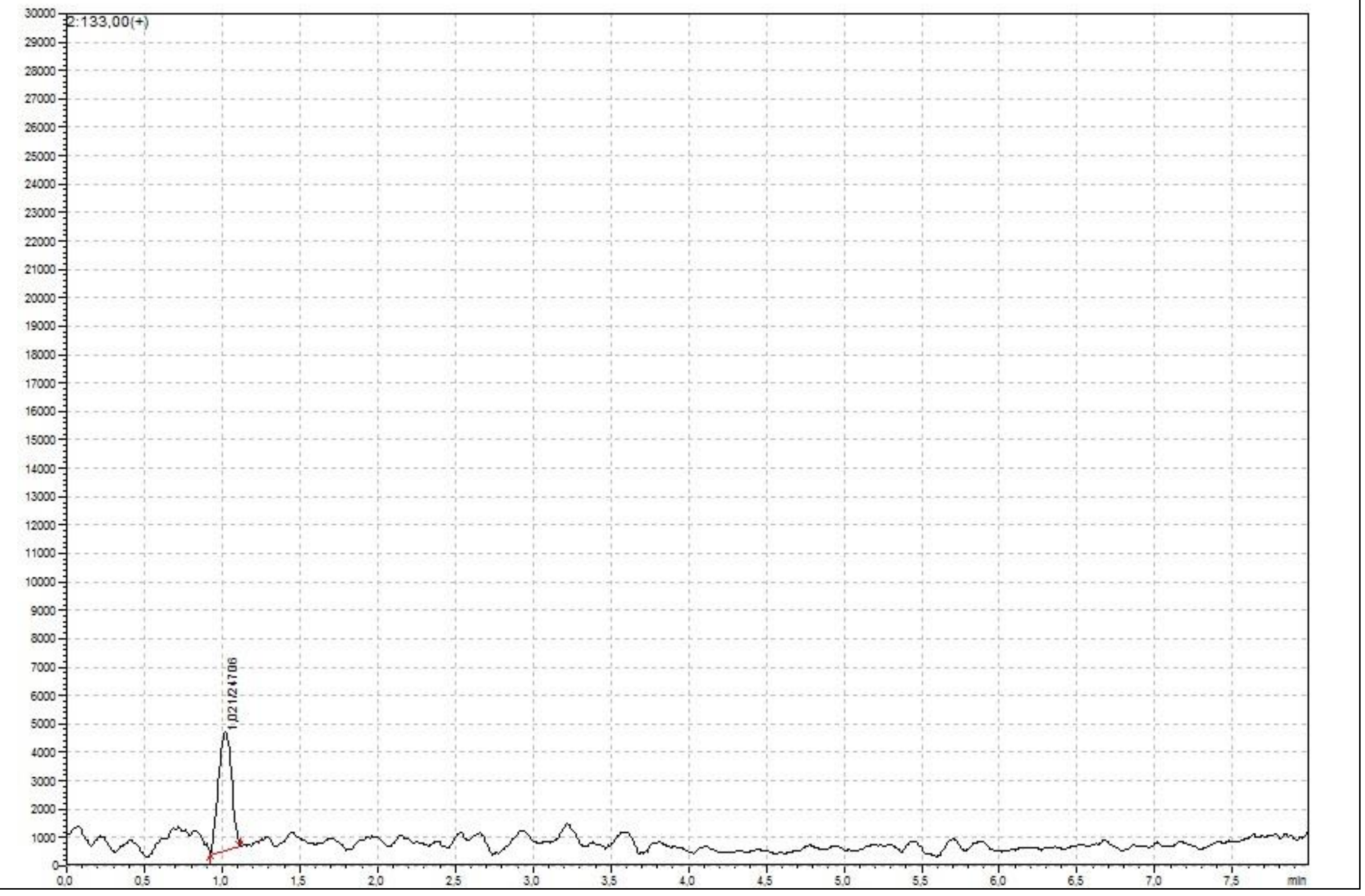
</td></tr>
</table>

Transformation product	Chromatogram
**TP23**<br>**Name:** 5-methyl-2,3-dihydro-1,2-oxazol-3-one<br>**SMILES:** CC1=CC(=O)NO1<br>**m/z:** 100 $[M+H]^+$<br>$\mathbf{t_R}$**:** 1.021 min	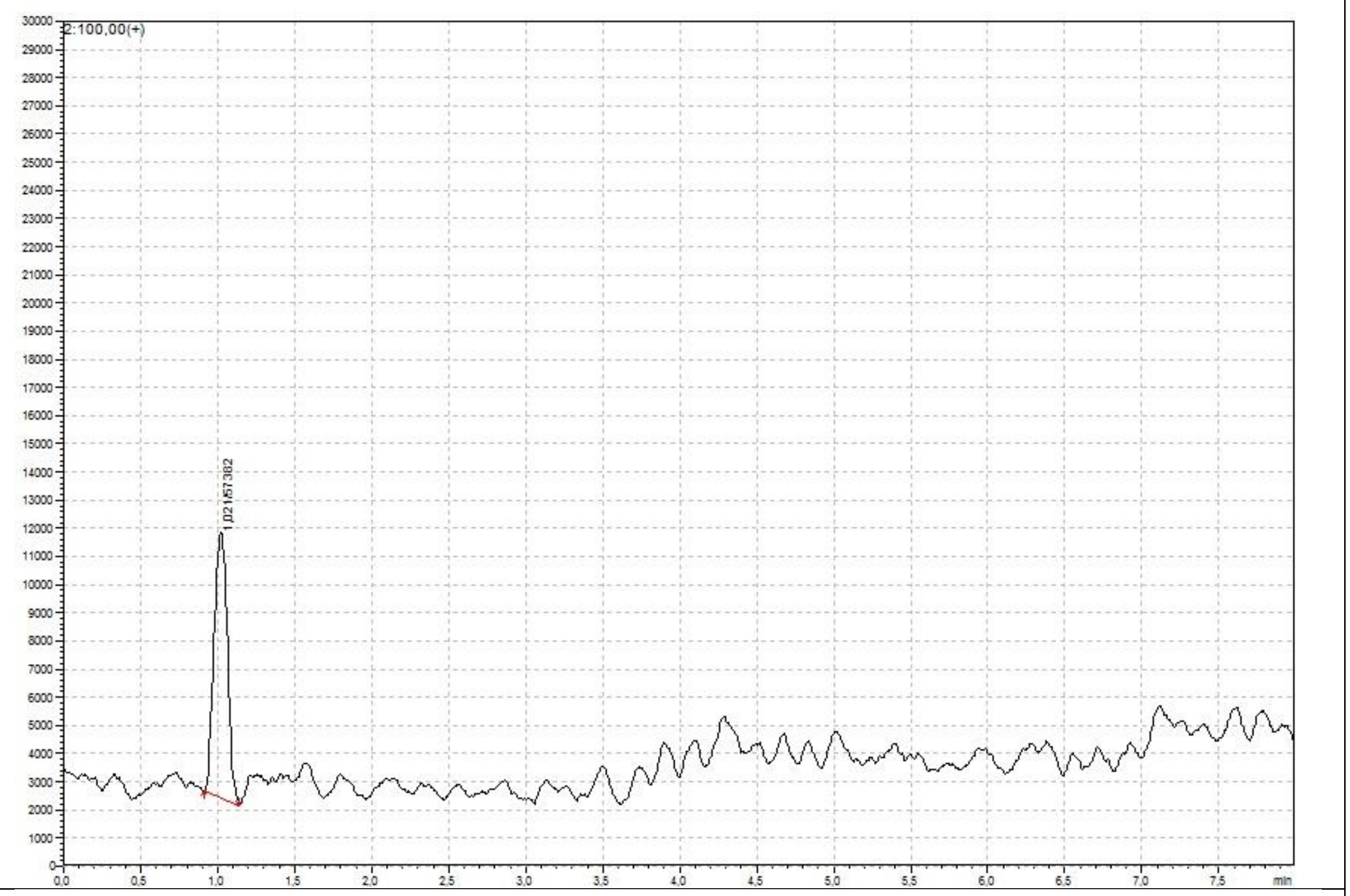
**TP24**<br>**Name:** N-(4-amino-3-chlorobenzenesulfonyl)carbamimidic acid<br>**SMILES:** NC1=C(Cl)C=C(C=C1)S(=O)(=O)NC(O)=N<br>**m/z:** 250 $[M+H]^+$ (^{35}Cl); 252 $[M+H]^+$ (^{37}Cl)<br>$\mathbf{t_R}$**:** 4.224 min	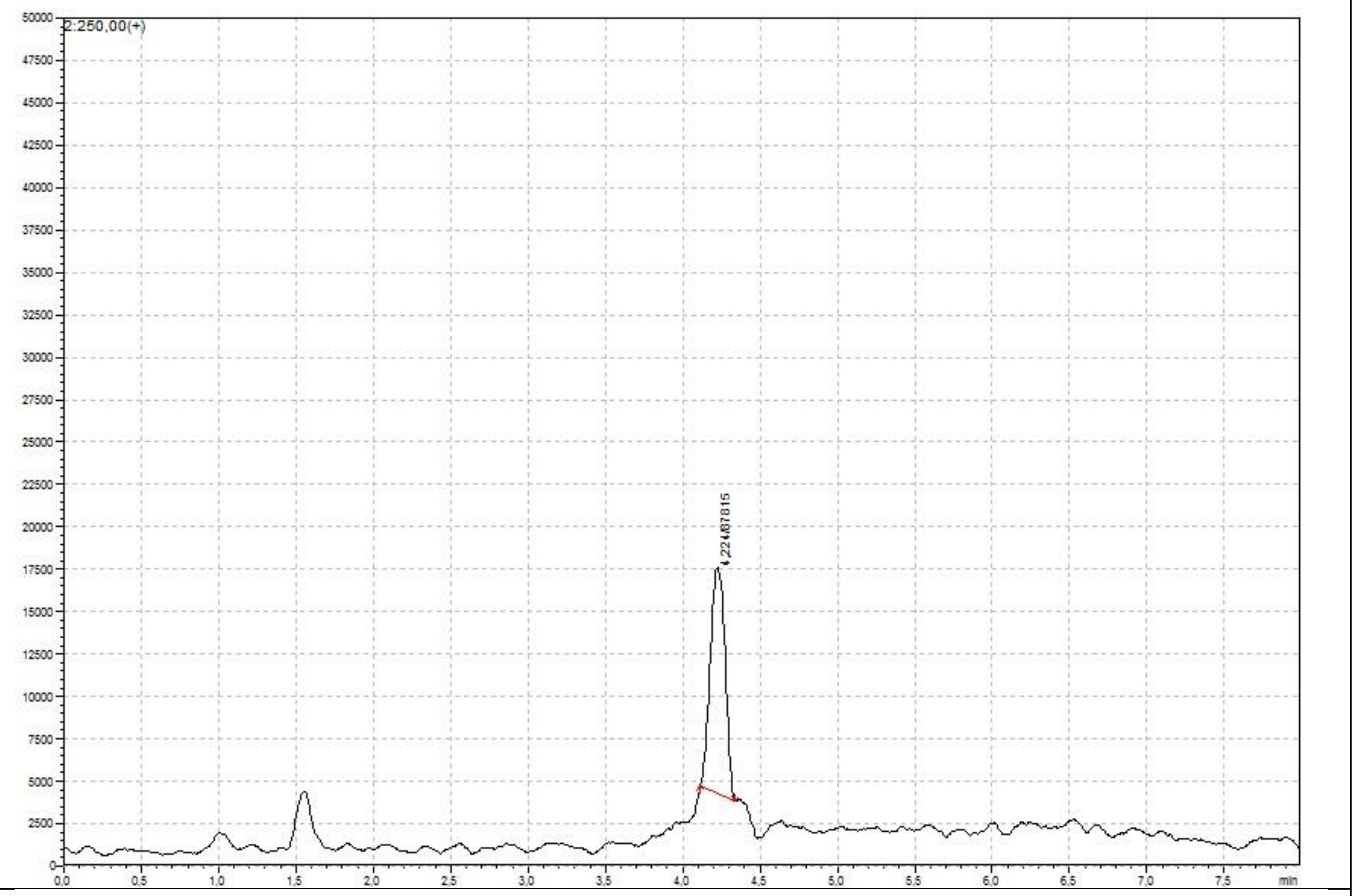
**TP25**<br>**Name:** 4-amino-2-chlorophenol<br>**SMILES:** NC1=CC(=C(O)C=C1)Cl<br>**m/z:** 143 $[M]^+$ (^{35}Cl); 145 $[M]^+$ (^{37}Cl)<br>$\mathbf{t_R}$**:** 1.024 min	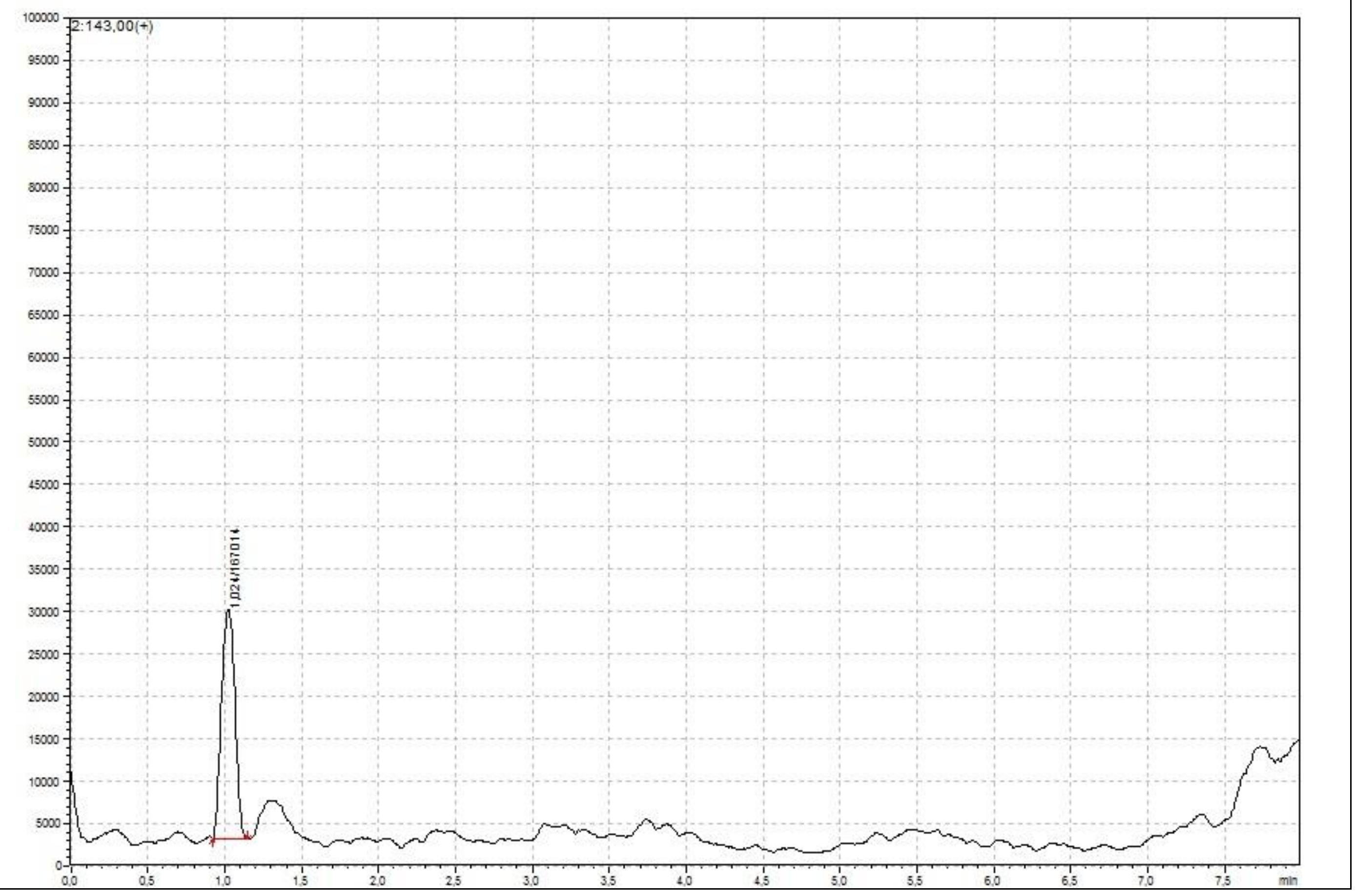

Transformation product	Chromatogram
**TP26**<br>**Name:** cyclohexa-2,5-diene-1,4-dione<br>**SMILES:** C1=CC(=O)C=CC1=O<br>**m/z:** 109 $[M+H]^+$<br>t_R: 1.033 min	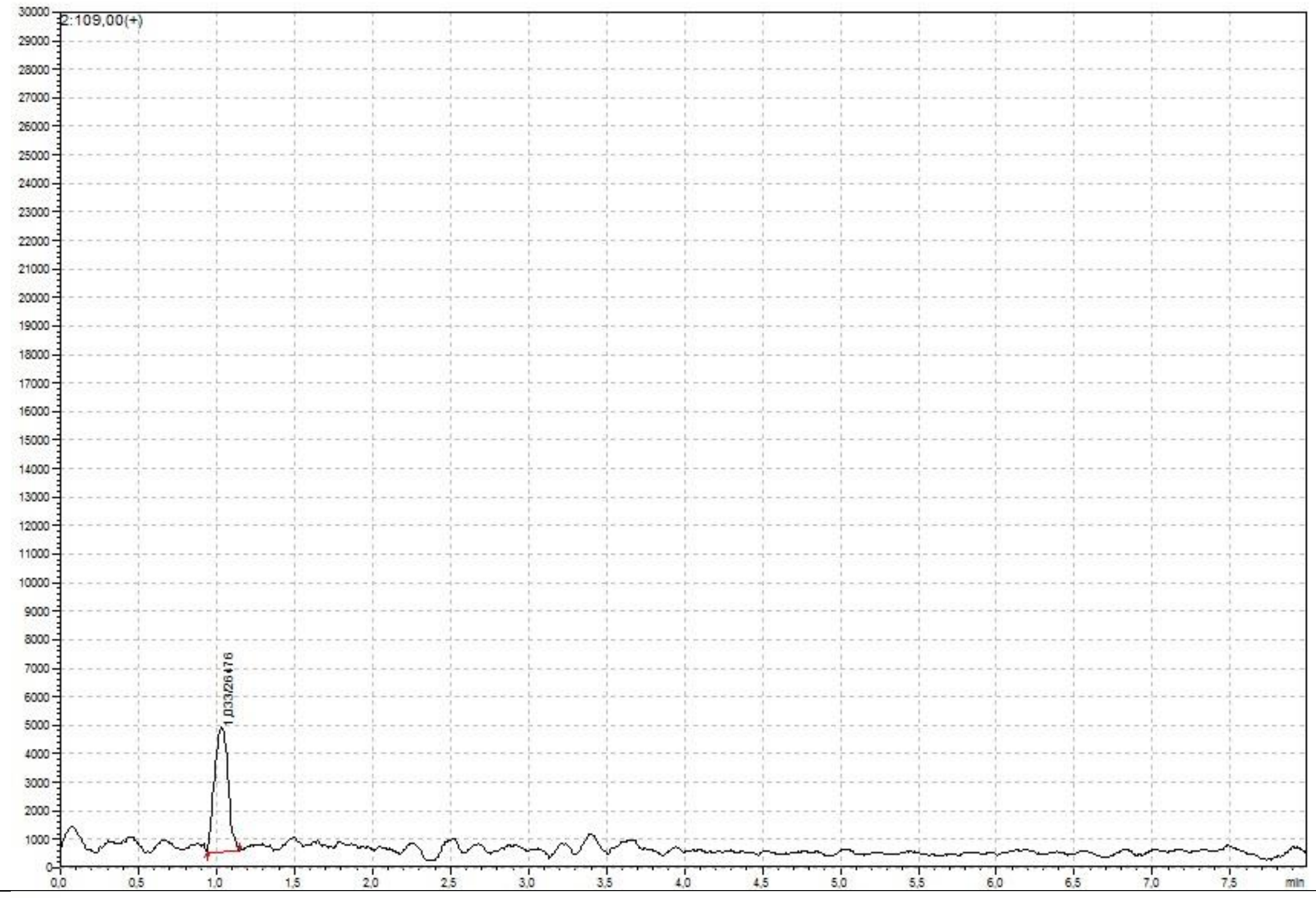
**TP27**<br>**Name:** 2-chloro-4-sulfonylaniline<br>**SMILES:** NC1=C(Cl)C=C(C=C1)S(=O)=O<br>**m/z:** 192 $[M+H]^+$ (^{35}Cl); 194 $[M+H]^+$ (^{37}Cl)<br>t_R: 3.720 and 3.905 min	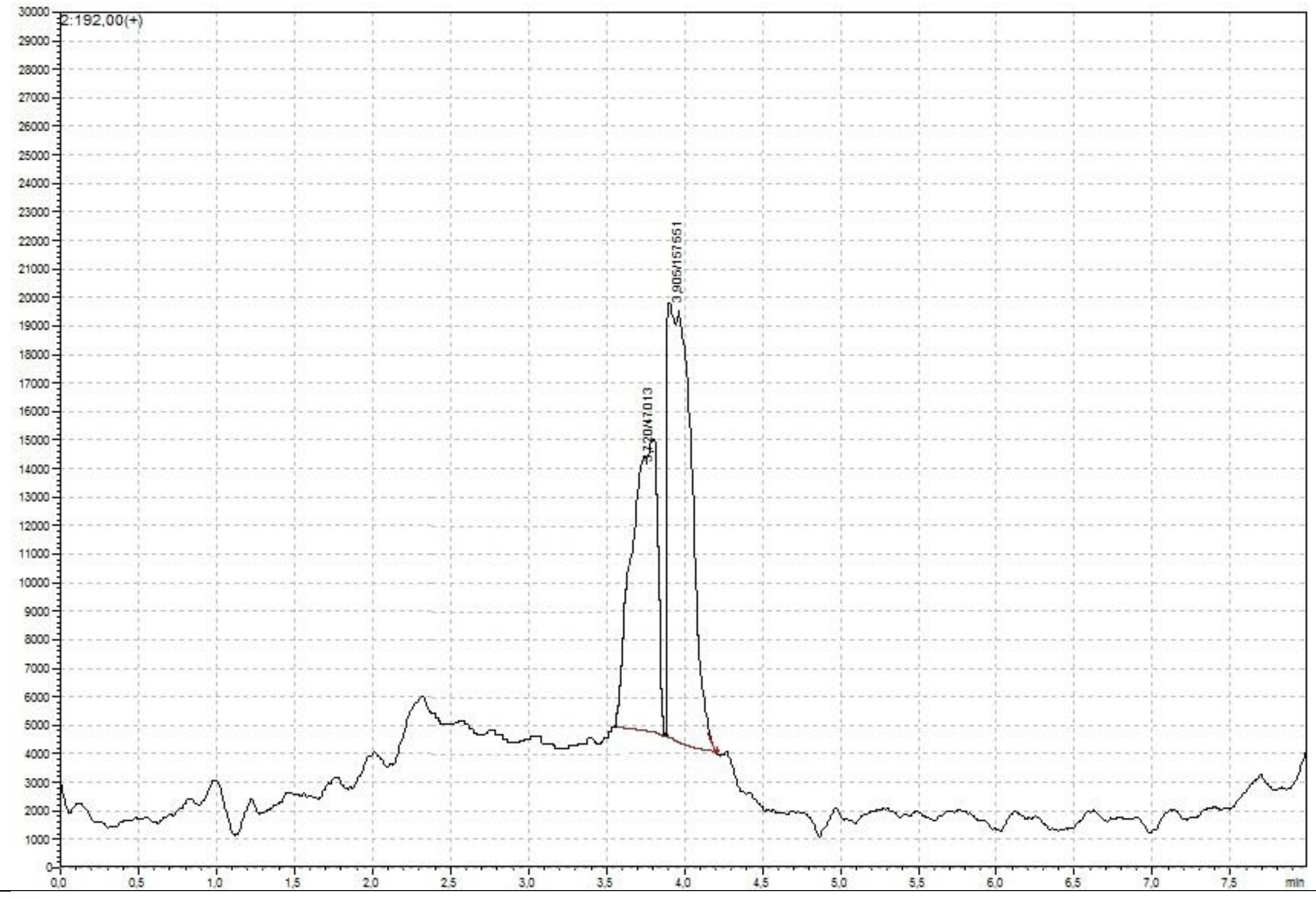
**TP28**<br>**Name:** (1S,2S,3S)-1-amino-S-(4-amino-3-chloro-5-hydroxyphenyl)-2,3-dihydroxybutane-1-sulfonamido<br>**SMILES:** C[C@H](O)[C@@H](O)[C@@H](N)NS(=O)(=O)C1=CC(Cl)=C(N)C(O)=C1<br>**m/z:** 327 $[M+H]^+$ (^{35}Cl); 329 $[M+H]^+$ (^{37}Cl)<br>t_R: 5.671 min	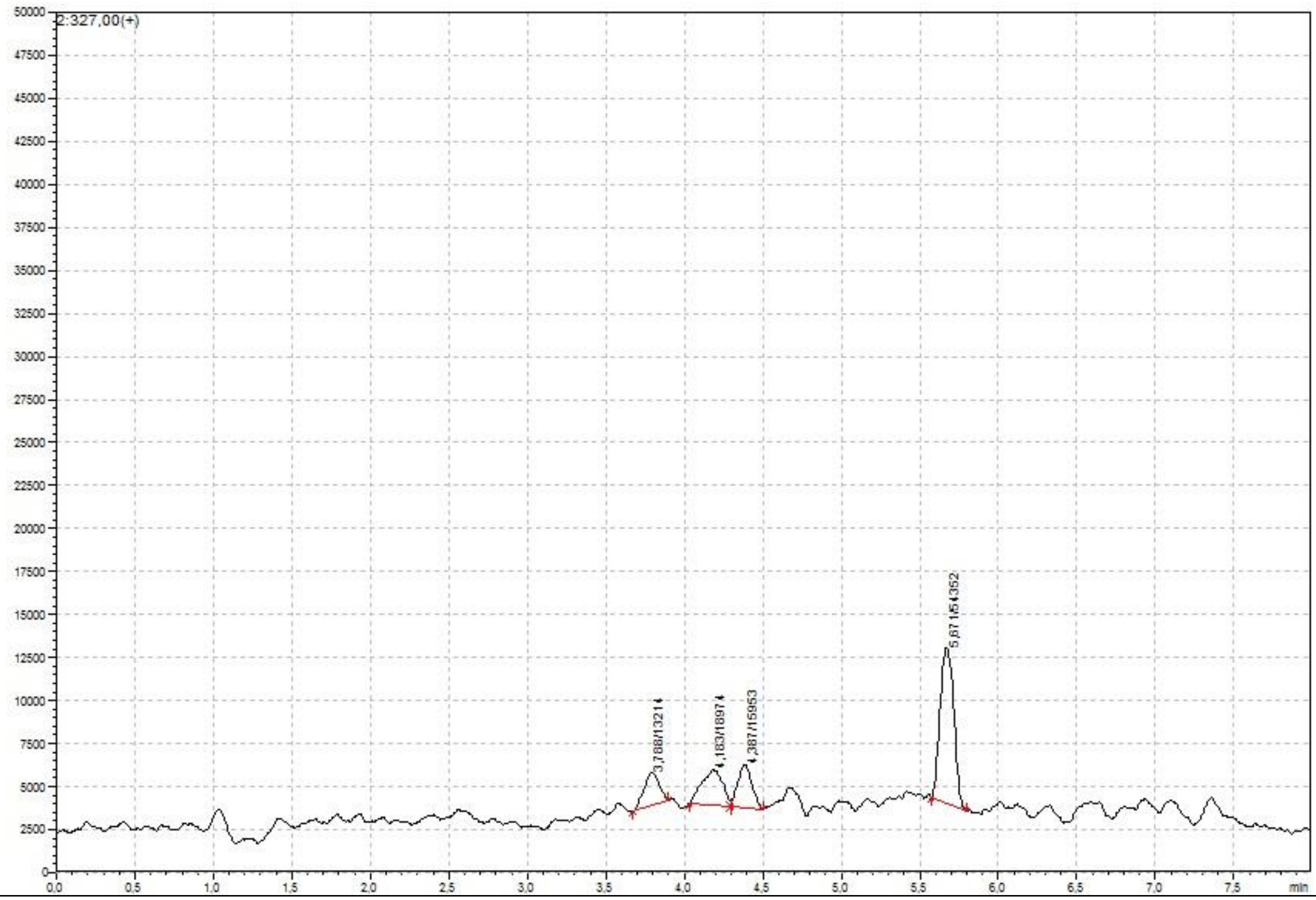

Transformation product	Chromatogram
**TP29**<br>**Name:** 3-chloro-N-(5-methyl-1,2-oxazol-3-yl)-4-nitrobenzene-1-sulfonamide<br>**SMILES:** CC1=CC(NS(=O)(=O)C2=CC=C(C(Cl)=C2)[N+]([O-])=O)=NO1<br>**m/z:** 318 $[M+H]^+$ (^{35}Cl); 320 $[M+H]^+$ (^{37}Cl)<br>$\mathbf{t_R}$: 3.813 min	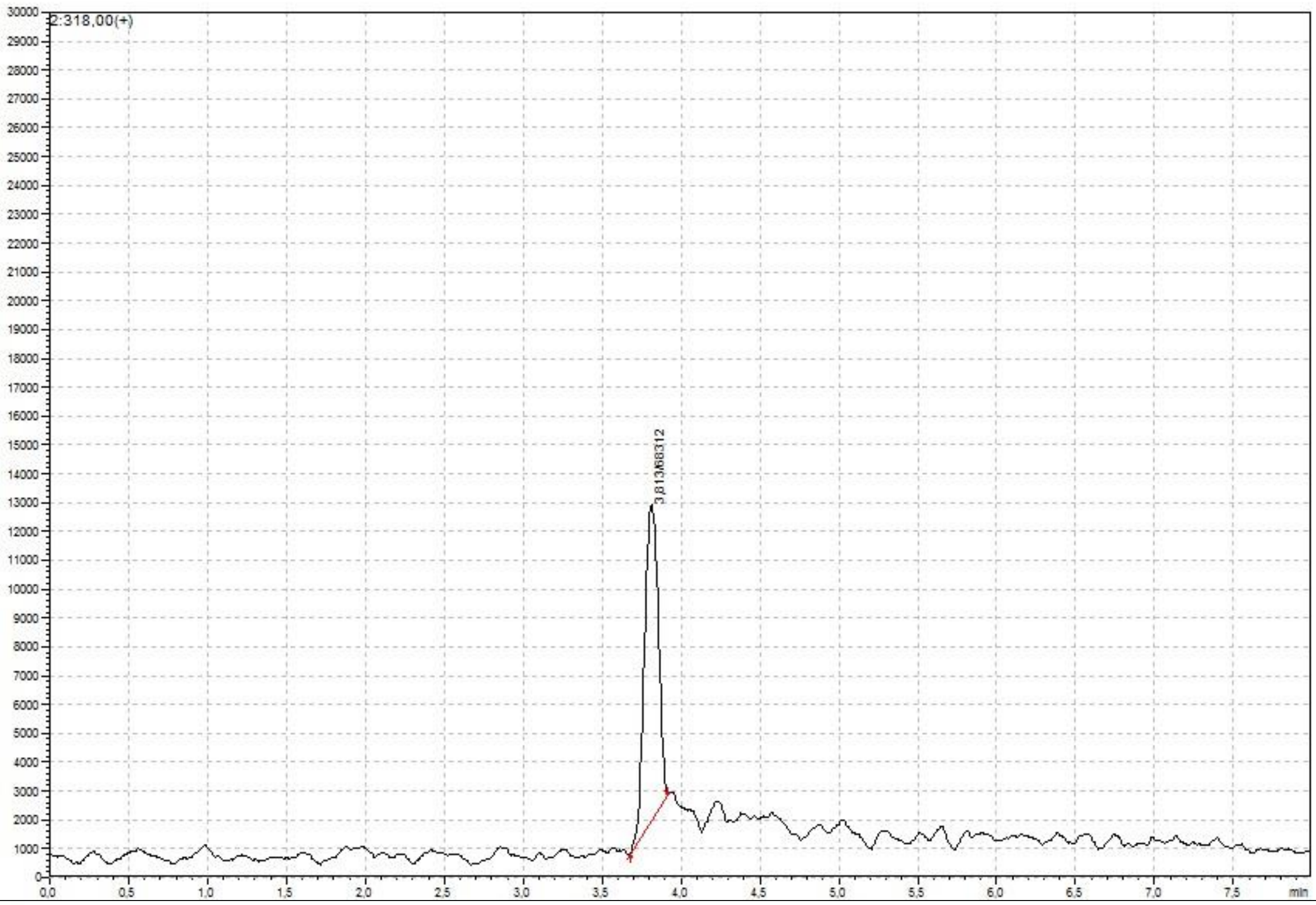
**TP30**<br>**Name:** 3-[2-(4-chloro-5-methyl-1,2-oxazol-3-yl)diazen-1-yl]-5-methyl-1,2-oxazol-4-ol<br>**SMILES:** CC1=C(O)C(N=NC2=NOC(C)=C2Cl)=NO1<br>**m/z:** 244 $[M+H]^+$ (^{35}Cl); 246 $[M+H]^+$ (^{37}Cl)<br>$\mathbf{t_R}$: 3.966 min	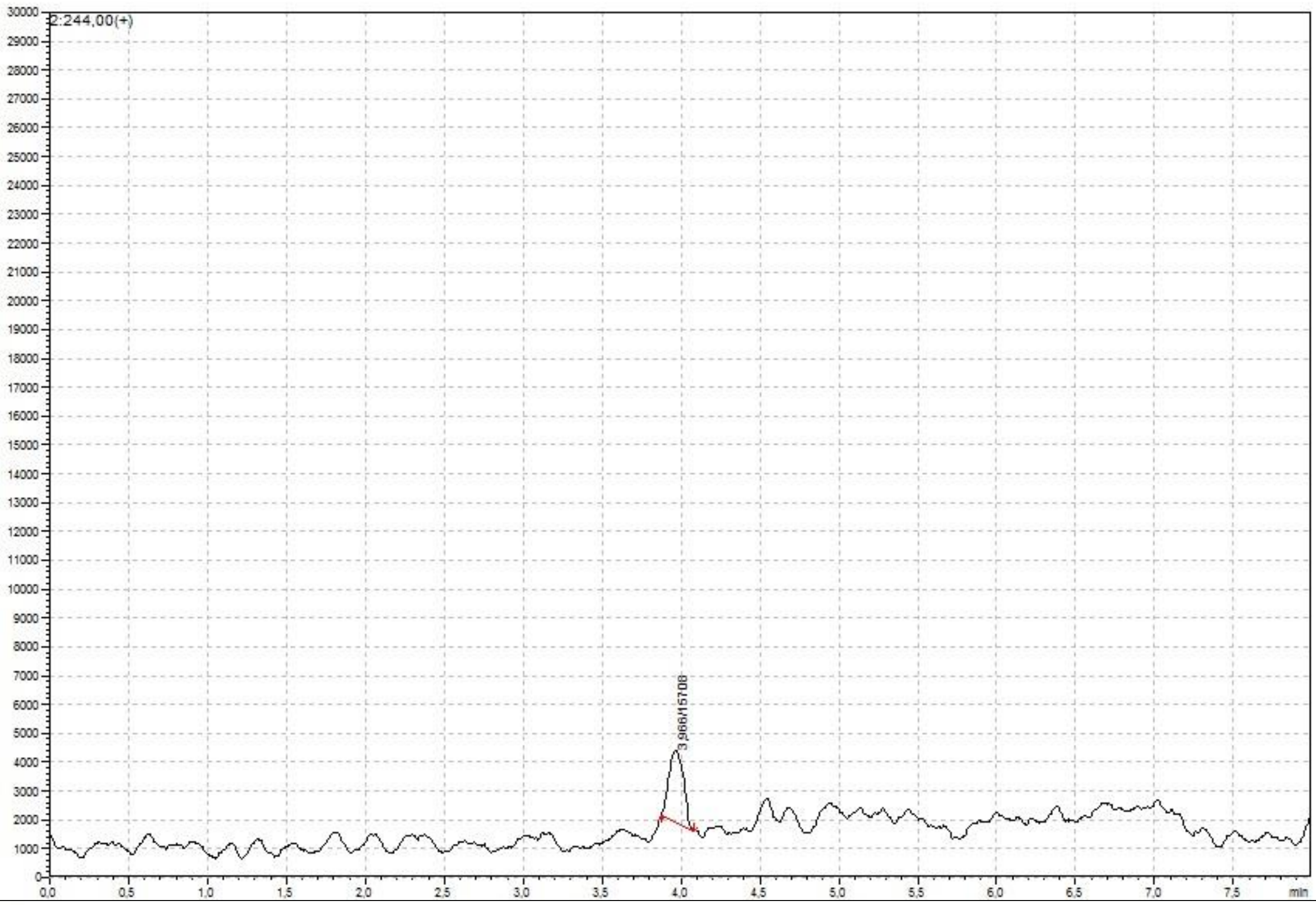
**TP31**<br>**Name:** 4-chloro-5-methyl-2,3-dihydro-1,2-oxazol-3-one<br>**SMILES:** CC=1ONC(=O)C=1Cl<br>**m/z:** 134 $[M+H]^+$ (^{35}Cl); 136 $[M+H]^+$ (^{37}Cl)<br>$\mathbf{t_R}$: 1.016 min	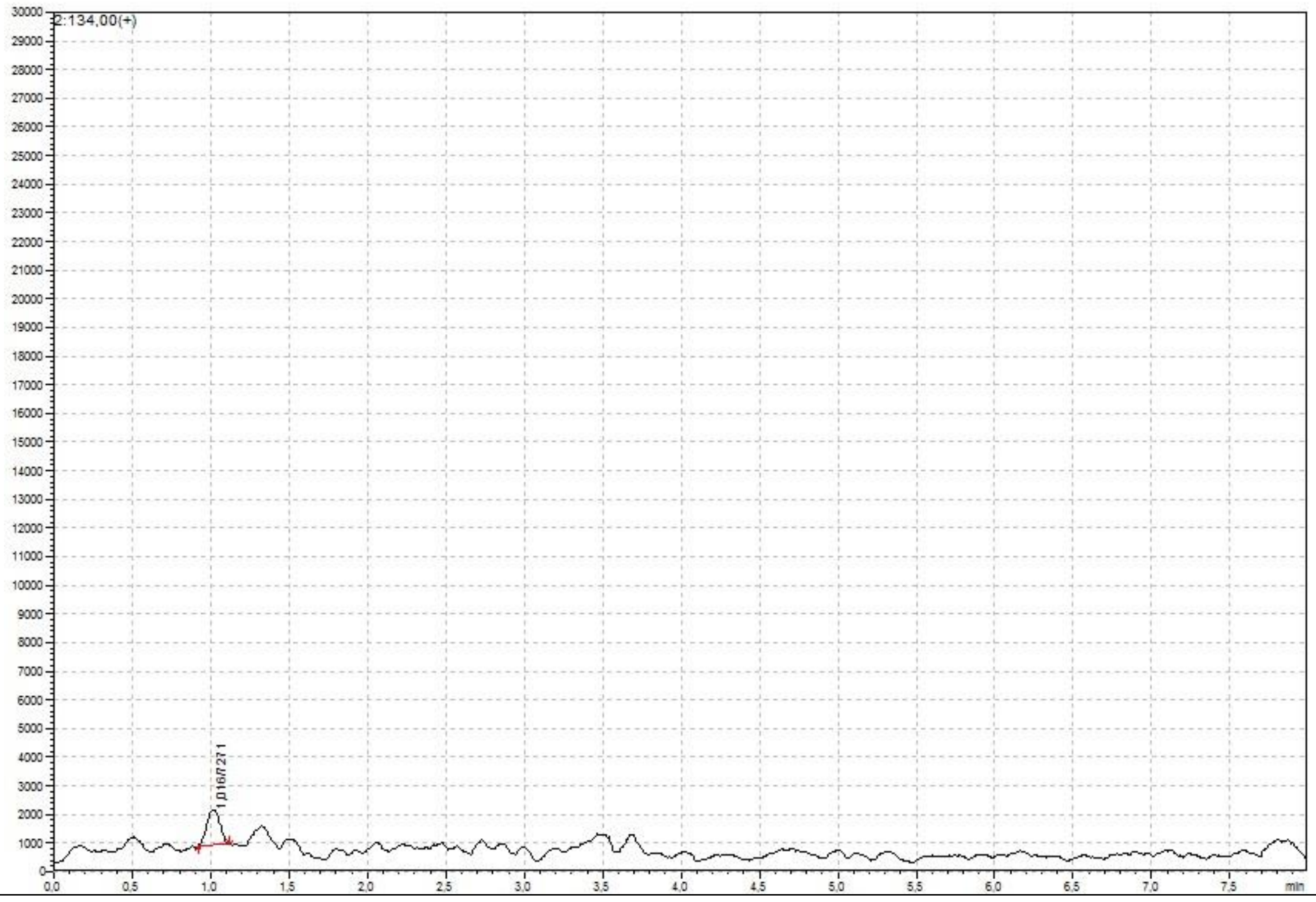

Transformation product	Chromatogram
**TP32**<br>**Name:** 2-chlorophenol<br>**SMILES:** OC1=CC=CC=C1Cl<br>**m/z:** 128 $[M]^+$ (^{35}Cl); 130 $[M]^+$ (^{37}Cl)<br>$\mathbf{t_R}$**:** 1.016 min	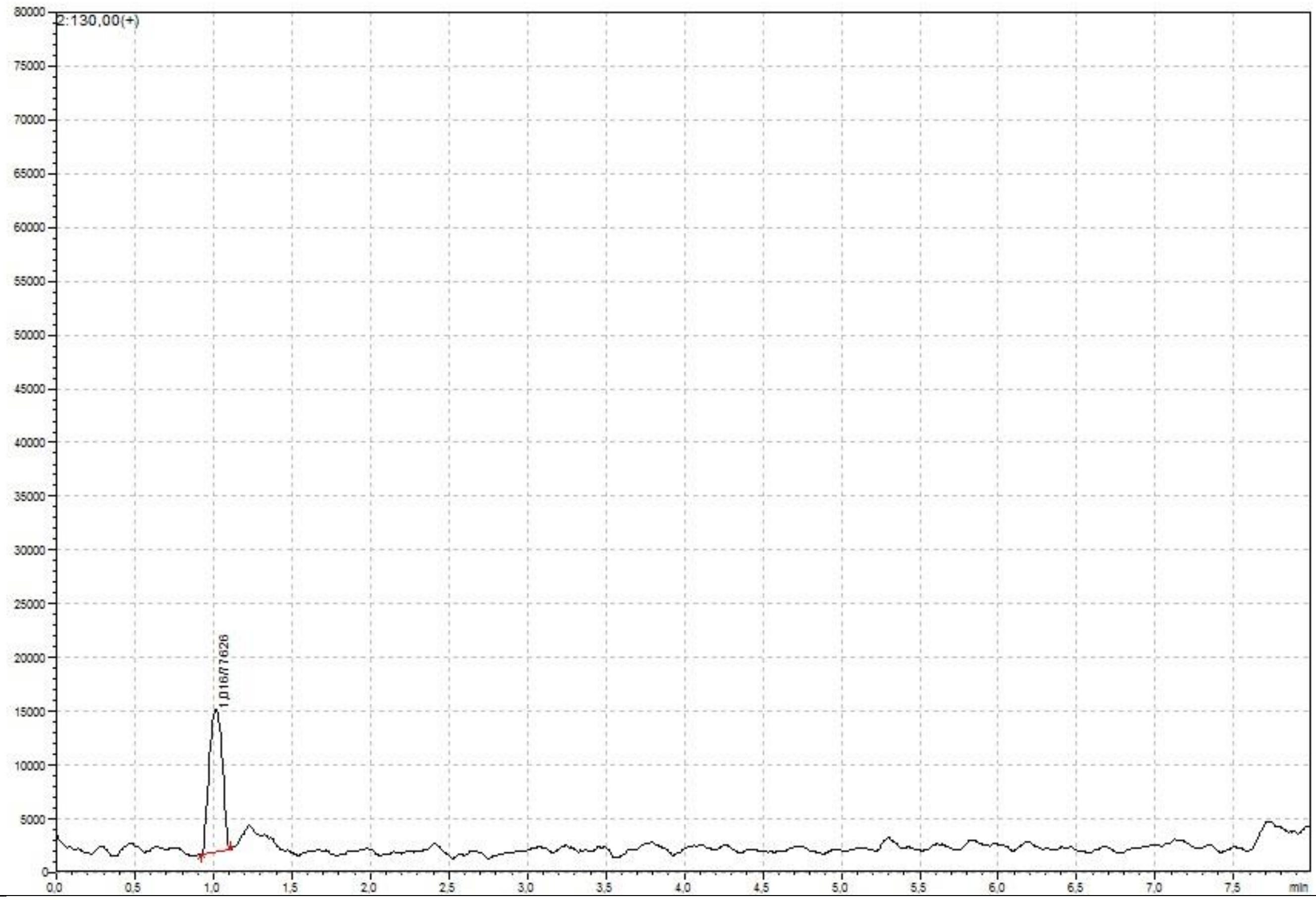
**TP33**<br>**Name:** 2-chlorocyclohexa-2,5-diene-1,4-dione<br>**SMILES:** ClC1=CC(=O)C=CC1=O<br>**m/z:** 143 $[M+H]^+$ (^{35}Cl); 145 $[M+H]^+$ (^{37}Cl)<br>$\mathbf{t_R}$**:** 1.024 min	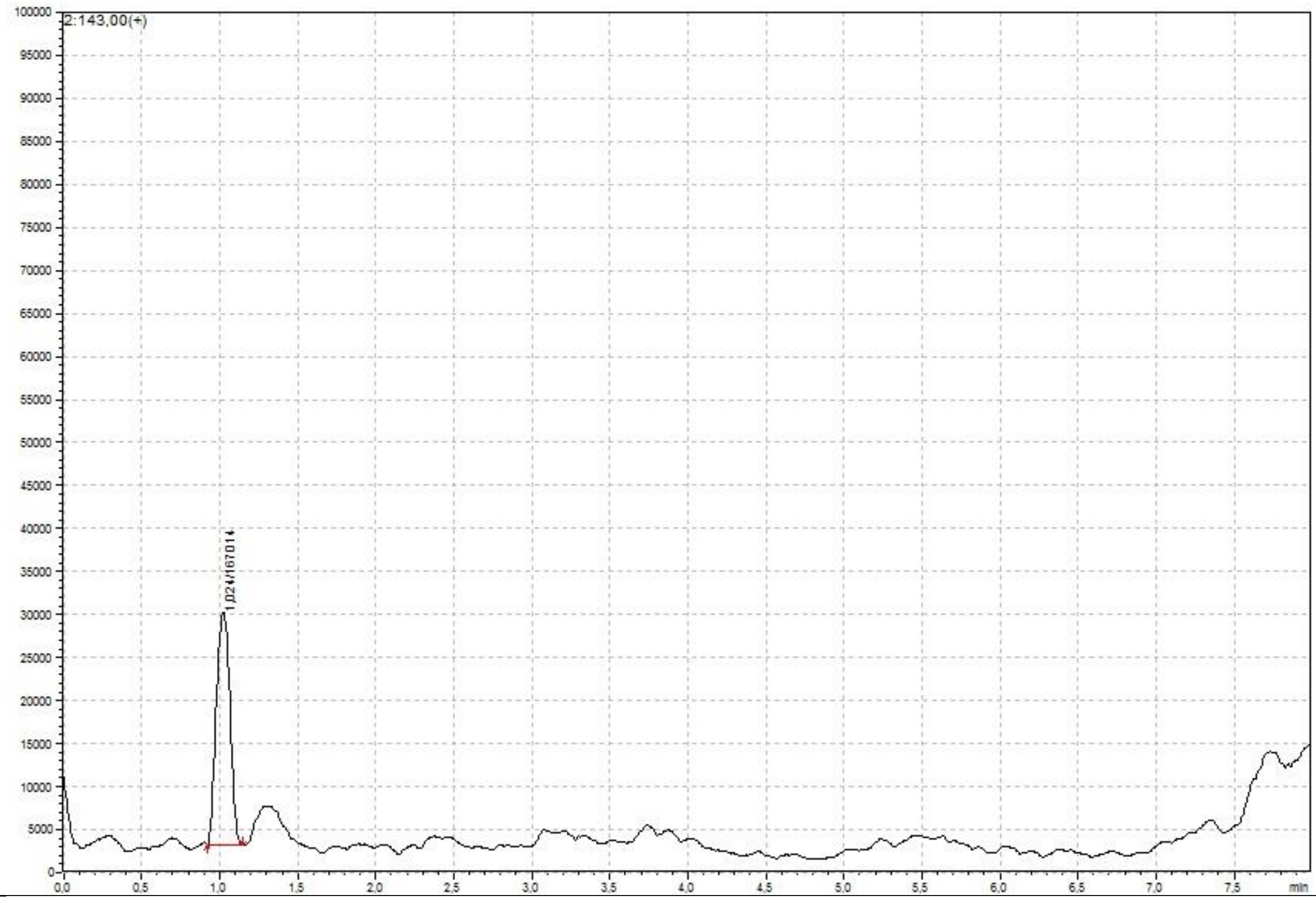
**TP34**<br>**Name:** 2-chloroaniline<br>**SMILES:** NC1=C(Cl)C=CC=C1<br>**m/z:** 127 $[M]^+$ (^{35}Cl); 129 $[M]^+$ (^{37}Cl)<br>$\mathbf{t_R}$**:** 1.075 min	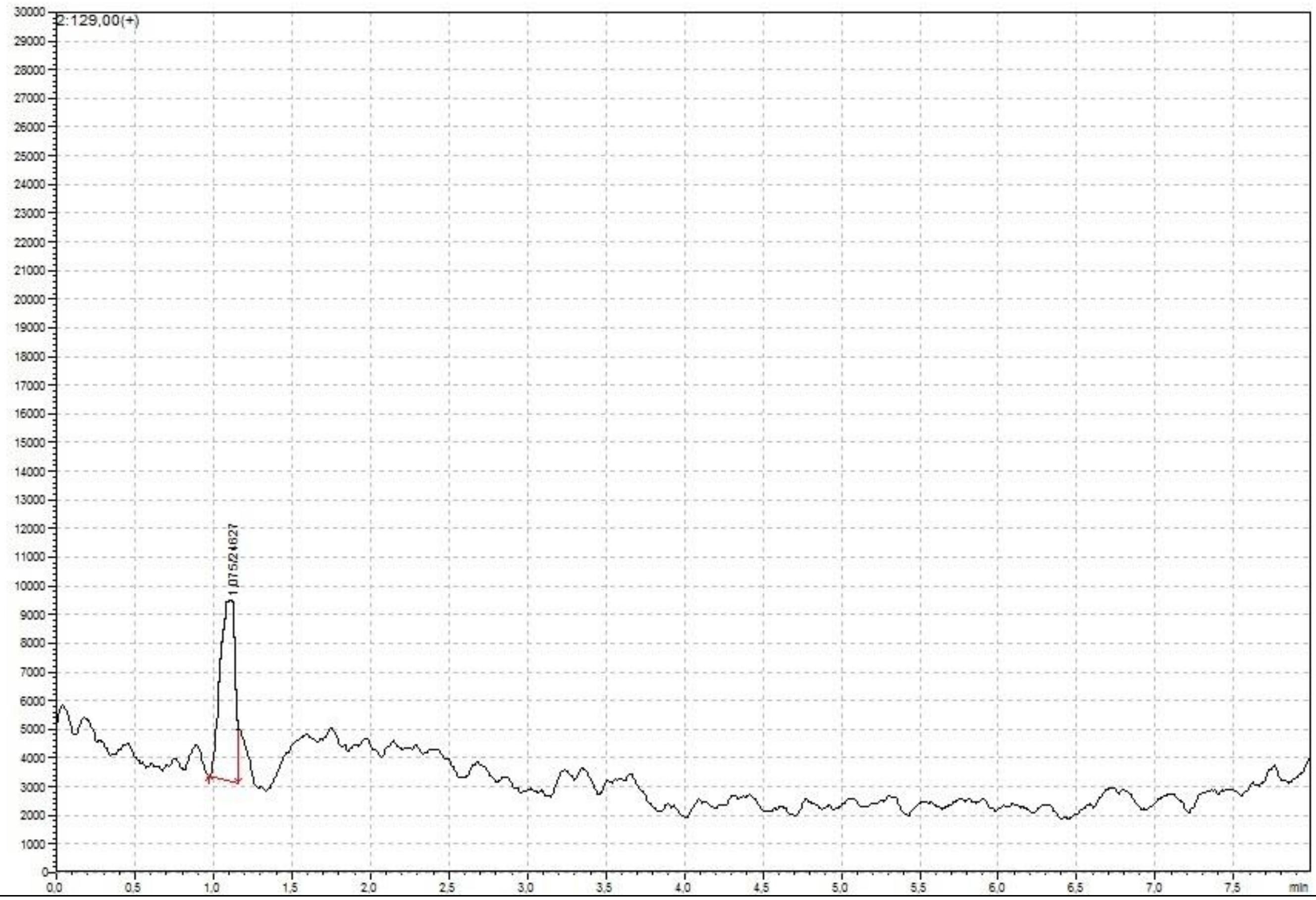
